# Supplementary material for: Cohort profile: the Western Cape Pregnancy Exposure Registry (WCPER)
Source: BMJ Open. 2022 Jun 29;12(6):e060205. doi: 10.1136/bmjopen-2021-060205 (PMC9244673; doi:10.1136/bmjopen-2021-060205)
Supplement: Supplementary data [file bmjopen-2021-060205supp001.pdf]

Supplementary Table 1. Potentially unsafe medicines, excluding ART, identified in the PER over the course of gestation (alphabetical)

**Name**
Carbamazepine
Carbimazole
Diazepam
Doxycycline
Enalapril
Gentamicin
Ibuprofen
Lithium
Losartan
Phenytoin
Sulfamethoxazole & trimethoprim
Valproate
Warfarin

ART – antiretroviral therapy
