# Supplementary material for: Cohort profile: the Western Cape Pregnancy Exposure Registry (WCPER)
Source: BMJ Open. 2022 Jun 29;12(6):e060205. doi: 10.1136/bmjopen-2021-060205 (PMC9244673; doi:10.1136/bmjopen-2021-060205)
Supplement: Author's manuscript [file bmjopen-2021-060205.draft_revisions.pdf]

BMJ Open is committed to open peer review. As part of this commitment we make the peer review history of every article we publish publicly available.

When an article is published we post the peer reviewers' comments and the authors' responses online. We also post the versions of the paper that were used during peer review. These are the versions that the peer review comments apply to.

The versions of the paper that follow are the versions that were submitted during the peer review process. They are not the versions of record or the final published versions. They should not be cited or distributed as the published version of this manuscript.

BMJ Open is an open access journal and the full, final, typeset and author-corrected version of record of the manuscript is available on our site with no access controls, subscription charges or pay-per-view fees (http://bmjopen.bmj.com).

If you have any questions on BMJ Open's open peer review process please email info.bmjopen@bmj.com

BMJ Open

Cohort Profile: The Western Cape Pregnancy Exposure Registry (WCPER)

Journal:	*BMJ Open*
Manuscript ID	bmjopen-2021-060205
Article Type:	Cohort profile
Date Submitted by the Author:	15-Dec-2021
Complete List of Authors:	Kalk, Emma; University of Cape Town Faculty of Health Sciences, Centre for Infectious Disease Epidemiology & Research<br>Heekes, Alexa; University of Cape Town Faculty of Health Sciences, Centre for Infectious Disease Epidemiology & Research; Western Cape Department of Health, Health Intelligence Directorate<br>Slogrove, Amy; Stellenbosch University, Ukwanda Centre for Rural Health, Department of Global Health; Stellenbosch University, Department of Paediatrics & Child Health<br>Phelanyane, Florence; University of Cape Town Faculty of Health Sciences, Centre for Infectious Disease Epidemiology & Research; Western Cape Department of Health, Health Intelligence Directorate<br>Davies, Mary-Ann; University of Cape Town Faculty of Health Sciences, Centre for Infectious Disease Epidemiology & Research; Western Cape Department of Health, Health Intelligence Directorate<br>Myer, Landon; University of Cape Town Faculty of Health Sciences, Division of Epidemiology & Biostatistics<br>Euvrard, Jonathan; University of Cape Town Faculty of Health Sciences, Centre for Infectious Disease Epidemiology & Research; Western Cape Department of Health, Health Intelligence Directorate<br>Kroon, Max; University of Cape Town Faculty of Health Sciences, Neonatology; Mowbray Maternity Hospital, Neonatal Services<br>Petro, Greg; University of Cape Town Faculty of Health Sciences, Department of Obstetrics & Gynaecology; New Somerset Hospital, Maternity Services<br>Fieggen, Karen; University of Cape Town Faculty of Health Sciences, Division of Human Genetics; Groote Schuur Hospital, Medical Genetics Services<br>Stewart, Chantal; University of Cape Town Faculty of Health Sciences, Department of Obstetrics & Gynaecology; Mowbray Maternity Hospital, Maternity Services<br>Rhoda, Natasha; University of Cape Town Faculty of Health Sciences, Department of Paediatrics and Child Health; Mowbray Maternity Hospital, Neonatal Services<br>Gebhardt, Stefan; Stellenbosch University, Department of Obstetrics & Gynaecology; Tygerberg Hospital, Maternity Services<br>Osman, Ayesha; University of Cape Town Faculty of Health Sciences, Department of Obstetrics & Gynaecology; Groote Schuur Hospital, Maternity Services<br>Anderson, Kim; University of Cape Town Faculty of Health Sciences, Centre for Infectious Disease Epidemiology & Research<br>Boulle, Andrew; University of Cape Town Faculty of Health Sciences,

	Centre for Infectious Disease Epidemiology & Research; Western Cape Department of Health, Health Intelligence Directorate<br>Mehta, Ushma; University of Cape Town Faculty of Health Sciences, Centre for Infectious Disease Epidemiology & Research

Keywords:	PUBLIC HEALTH, EPIDEMIOLOGY, Maternal medicine < OBSTETRICS, Fetal medicine < OBSTETRICS

SCHOLARONE™
Manuscripts

I, the Submitting Author has the right to grant and does grant on behalf of all authors of the Work (as defined in the below author licence), an exclusive licence and/or a non-exclusive licence for contributions from authors who are: i) UK Crown employees; ii) where BMJ has agreed a CC-BY licence shall apply, and/or iii) in accordance with the terms applicable for US Federal Government officers or employees acting as part of their official duties; on a worldwide, perpetual, irrevocable, royalty-free basis to BMJ Publishing Group Ltd ("BMJ") its licensees and where the relevant Journal is co-owned by BMJ to the co-owners of the Journal, to publish the Work in this journal and any other BMJ products and to exploit all rights, as set out in our licence.

The Submitting Author accepts and understands that any supply made under these terms is made by BMJ to the Submitting Author unless you are acting as an employee on behalf of your employer or a postgraduate student of an affiliated institution which is paying any applicable article publishing charge ("APC") for Open Access articles. Where the Submitting Author wishes to make the Work available on an Open Access basis (and intends to pay the relevant APC), the terms of reuse of such Open Access shall be governed by a Creative Commons licence – details of these licences and which Creative Commons licence will apply to this Work are set out in our licence referred to above.

Other than as permitted in any relevant BMJ Author's Self Archiving Policies, I confirm this Work has not been accepted for publication elsewhere, is not being considered for publication elsewhere and does not duplicate material already published. I confirm all authors consent to publication of this Work and authorise the granting of this licence.

For peer review only

Cohort Profile: The Western Cape Pregnancy Exposure Registry (WCPER)

Authors: Emma Kalk[1*], Alexa Heekes[1,2,] Amy Slogrove[3,4], Florence Phelanyane[1,2], Mary-Ann Davies[1,2], Landon Myer[5], Jonathan Euvrard[1,2], Max Kroon[6,7], Greg Petro[8,9], Karen Fieggen[10,11], Chantal Stewart[8,12], Natasha Rhoda[6,7], Stefan Gebhardt[13,14], Ayesha Osman[8,15], Kim Anderson[1], Andrew Boulle[1,2†], Ushma Mehta[1†]

1. Centre for Infectious Disease Epidemiology & Research and Division of Public Health Medicine, School of Public Health & Family Medicine, University of Cape Town, South Africa

2. Health Intelligence Directorate, Western Cape Government Health, Cape Town, South Africa

3. Department of Paediatrics & Child Health, Faculty of Medicine & Health Sciences, Stellenbosch University, Worcester, South Africa

4. Ukwanda Centre for Rural Health, Department of Global Health, Faculty of Medicine & Health Sciences, Stellenbosch University, Worcester, South Africa

5. Division of Epidemiology & Biostatistics, School of Public Health & Family Medicine, University of Cape Town, South Africa

6. Department of Paediatrics and Child Health, University of Cape Town, South Africa

7. Mowbray Maternity Hospital, Neonatal Services, Cape Town, South Africa

8. Department of Obstetrics & Gynaecology, University of Cape Town, South Africa

9. New Somerset Hospital, Maternity Services, Cape Town, South Africa

10. Division of Human Genetics, University of Cape Town, Cape Town, South Africa

11. Groote Schuur Hospital, Medical Genetics Service, Cape Town, South Africa

12. Mowbray Maternity Hospital, Maternity Services, Cape Town, South Africa

13. Department of Obstetrics & Gynaecology, Stellenbosch University, Cape Town, South AFrica

For peer review only

14. Tygerberg Hospital Maternity Services, Cape Town, South Africa

15. Groote Schuur Hospital, Maternity Services, Cape Town South Africa

*corresponding author: Centre for Infectious Disease Epidemiology & Research, School of Public Health & Family Medicine, 5th floor Falmouth Building, University of Cape Town Faculty of Health Sciences, Anzio Road, Observatory, Cape Town

emma.kalk@uct.ac.za

† Joint senior authors

For peer review only

ABSTRACT

Purpose: The Western Cape Pregnancy Exposure Registry (PER) was established at two public sector healthcare sentinel sites in the Western Cape province, South Africa to provide on-going surveillance of drug exposures in pregnancy and associations with pregnancy outcomes.

Participants: Established in 2016, all women attending their first antenatal visit at primary care obstetric facilities were enrolled and followed to pregnancy outcome regardless of the site (i.e., primary, secondary, tertiary facility). Routine operational obstetric and medical data are digitized from the clinical stationery at the health care facilities. Data collection has been integrated into existing services and information platforms and supports routine operations. The PER is situated within the Provincial Health Data Centre, an information exchange that harmonizes and consolidates all health-related electronic data in the province. Data are contributed via linkage across a unique identifier. This relationship limits the missing data in the PER, allows validation and avoids misclassification in the population-level dataset.

Findings to date: Approximately 5000 and 3500 pregnant women enter the dataset annually at the urban and rural sites, respectively. As of August 2021, >30 000 pregnancies have been recorded and outcomes have been determined for 93%. Analysis of key obstetric and neonatal health indicators derived from the PER are consistent with the aggregate data in the District Health Information System.

Future plans: This represents significant infrastructure, able to address clinical and epidemiological concerns in a low/middle-income setting.

Key words

Pregnancy Exposure Registry, Pharmacovigilance, Surveillance

For peer review only

Strengths and Limitations of this Study

The Western Cape Pregnancy Exposure Registry (PER) was established to provide on-going surveillance of drug exposures in pregnancy and associations with pregnancy outcomes. The system comprises unique infrastructure able to address clinical and public health concerns in a low-middle income setting.

Data collection has been integrated into existing services and information platforms and supports routine operations.

The PER is situated within the Provincial Health Data Centre, an information exchange that harmonizes and consolidates all health-related electronic data in the province; this relationship limits the missing data in the PER, allows validation and avoids misclassification in the population-level dataset.

The PER digitizes clinical data recorded in operational stationery and we cannot control for data quality nor account for missing data in the source documents and unmeasured confounders.

Medicines obtained outside the public sector systems and traditional and complementary medicines are not included unless they are documented in the clinical stationery.

For peer review only

Cohort Profile: The Western Cape Pregnancy Exposure Registry

Introduction

Assessing medicine and vaccine safety in pregnancy requires on-going surveillance across multiple settings. In high-income countries, reviews of outpatient prescriptions and self-medication during pregnancy estimated exposure rates of up to 93% and 43%, respectively, excluding vitamins and supplements[1, 2]. Reports from Africa, the site of mass prevention and treatment campaigns for HIV, tuberculosis and malaria, are less frequent: we estimate that 79% - 99% of women in Cape Town use medicines antenatally[3].

Pregnant women have been systematically excluded from pharmaceutical trials and the efficacy, dosing and safety of many medicines used during pregnancy are uncertain. Post-authorization safety assessments have traditionally relied on passive reporting of suspected medicine-related adverse events. Such systems are limited by their dependence on voluntary reporting, variable data quality, absence of background rates of adverse birth outcomes including common congenital disorders, and lack of data to establish a denominator.

Recently, pharmacovigilance in pregnancy has drawn public and political attention following concerns about the association between the antiretroviral integrase inhibitor, dolutegavir, and neural tube defects[4, 5], the potential risk of isoniazid preventive therapy in women living with HIV[6] (WLHIV), and SARS-CoV-2 vaccines[7].

Pregnancy Exposure Registries (PER) are a form of surveillance, designed to iteratively detect adverse events within a defined pregnant population. Importantly, the prospective nature of PER allows collection of exposure and other data before the pregnancy outcome is known. The pharmaceutical

For peer review only

industry maintains drug-specific registries for medicines and/or drug classes with known/suspected teratogenic effects or as part of post-marketing commitments; e.g., the Antiretroviral Pregnancy and Anti-Epileptic Drug Registries[8, 9]. In addition, teratology information services may collect data on pregnancy exposures. These PER depend on voluntary enrolment by clinicians and/or women, and many do not directly collect data from comparator groups but rely either on internal comparators or on an identified external comparator to provide background prevalence data[10]. Background rates of adverse maternal and obstetric outcomes are necessary to determine deviations from expected proportions (signals). Such data may be limited or lacking in low- and middle-income countries[11, 12] or differ sufficiently from the source population so as to introduce bias (e.g., use of the Metropolitan Atlanta Congenital Defects Program as external comparator for USA-based studies[10].)

The World Health Organization (WHO) has developed a PER approach for resource-limited settings aimed at prospective data collection on exposures in a cohort of pregnant women attending antenatal care services at sentinel sites. Important for validity and causality determination, the approach recommends inclusion of *all* women presenting to the site to allow concurrent establishment of background rates and assessment of multiple potential exposures[13].

The Western Cape (WC) PER was established in Cape Town in 2016, adapted from the WHO template. It was nested within the province-wide health information exchange, a component within a larger project designed to assess the impact of WHO Option B+ for vertical HIV transmission prevention (i.e., universal lifelong antiretroviral therapy [ART] for pregnant and breast-feeding women) at the population and individual levels[14]. Situating the PER within the linked information exchange avoided some of the limitations of exclusive primary-care databases in that both electronic inpatient and outpatient prescriptions are recorded as well as those from specialist and other off-site clinics, sources which may be absent from primary-care records[3, 15, 16]. The design also supports augmentation of

For peer review only

the electronic clinical record for enrolled women, while providing a more secure, sustainable, and ethically-viable platform for capturing clinical data on mothers and infants.

We took a pragmatic approach to the establishment of the PER based on the availability of resources and the desire to integrate into existing systems and operational routines, avoiding a parallel infrastructure and supporting longevity. Data generated by the initiative are available for the evaluation and improvement of clinical care as well as epidemiological review.

Cohort description

The PER has been established at two sentinel sites in the WC. Gugulethu Midwife Obstetric Unit (GMOU) provides obstetric care to approximately 5000 women annually in Gugulethu, Cape Town a low-income area with high unemployment and an antenatal HIV prevalence of approximately 30%. GMOU refers patients to Mowbray Maternity (secondary) and Groote Schuur (tertiary) Hospitals. About half of all women who attend GMOU are referred to hospital, antenatally or perinatally. Worcester MOU (WMOU) is situated adjacent to the Worcester Provincial Hospital in Worcester, a town of approximately 230 000 in a farming community 120 kilometers outside Cape Town. WMOU provides delivery services for ~3600 women annually. The antenatal HIV prevalence is approximately 16%. Women requiring more advanced care are referred to Worcester (secondary) and Tygerberg (tertiary) Hospitals. The community is structurally disadvantaged, and many depend on seasonal employment on farms. In both areas the population is mobile; women move within the WC province and may deliver outside the proscribed referral axes.

Enrolment started at GMOU in Cape Town in September 2016 and at WMOU in January 2018.

For peer review only

All women seeking care at the sentinel primary-care sites were included. Most women who use public maternity services, including those with medical and obstetric complications, initially present to primary care, therefore **situating enrolment at the primary-care facility allowed us to capture a sample representative of the pregnant population in the geographic drainage area of that facility.**

Maternal and Child Health Services in the Western Cape

Obstetric care is free at the point of service and approximately 65% of women present at/before 20 weeks gestation[17]. Antenatal care for uncomplicated pregnancies is provided at Basic Antenatal Clinics and MOU, the latter able to manage uncomplicated vaginal deliveries. At any stage during pregnancy or peri-partum women can be referred to district, regional or tertiary hospitals according to standard operating procedures. HIV testing is routine at timepoints throughout gestation and WLHIV are initiated/re-initiated on ART[18]; those already receiving ART may transfer their HIV care to the MOU. Clients with other underlying medical conditions (e.g., pre-existing hypertension, diabetes mellitus, cardiac conditions) and/or who develop pregnancy-related medical conditions (e.g., hypertensive disorders of pregnancy, gestational diabetes) continue antenatal care at hospital. The MOU dispenses ART and antenatal supplements and preventive therapies recommended by the WHO in pregnancy (i.e. iron and folate supplements, tetanus and influenza vaccines)[19]. Midwives treat the common complaints of pregnancy (heartburn, nausea), urinary tract infection, vaginal candidiasis and provide syndromic treatment for sexually transmitted infections (STI). Frequently, these medicines are dispensed directly from *ward-stock* without a linked digital record, although details are recorded in paper-based registers.

For peer review only

Within resource constraints, the WC endeavors to provide an antenatal ultrasound scan to clients before 22 weeks gestation for determining gestational age. If concerns are identified women are up-referred for formal fetal anomaly review.

Antenatal visits, HIV testing, transfers and deliveries are recorded against patient names in individual paper-based registers. Monthly aggregate statistics of key obstetric indicators (Table 1) are manually counted from these registers and submitted centrally as part of the routine District Health Information System platform.

Follow-up

The Maternity Case Record (MCR) is a patient-held paper-based document distributed at the first antenatal visit that serves as a record of all clinical obstetric care until discharge after pregnancy outcome, regardless of level of care. It is utilized throughout South Africa and archived at the site of outcome. Chronic medication and any agents dispensed during pregnancy should be recorded in the MCR by the attending clinicians. However, medicines received at specialist clinics, during hospital admissions and over-the-counter medicines are often not documented [3, 15].

After birth, live- and still-born neonates are examined by the attending clinician (nurse midwife/doctor) and the outcome of the limited neonatal surface examination is recorded in the MCR. This examination has been shown to detect most major congenital malformations in neonates, i.e. those that are visible and do not require diagnostic tools[20]. At GMOU, a clinician employed by the PER performs a review of clinical records to obtain additional data for congenital disorders and stillbirths. In the case of stillbirth, the placenta may be sent for histological examination.

For peer review only

In the WC, most women (99%) give birth at a health facility[17]. Those who do not, will bring their infants to the MOU soon after birth for review and registration.

For the purposes of the PER, the MCR serves as the primary source of prospectively-collected clinical data. Thus, women enter the cohort on first visit to the MOU and are followed up until pregnancy outcome.

Data collection

The PER digitizes routinely-collected data from the clinical stationery if not already digitized under existing service delivery. In addition to the patient-held MCR, data sources include primary-care dating ultrasound reports, and the STI and labour ward delivery registers. As we are using operational data, definitions have been aligned with operational clinical definitions in the WC. Using other routinely collected data elements (gestational age, neonate anthropometry) we are able to align case definitions with those of the Global Alignment of Immunization Safety Assessment in Pregnancy[21], allowing for harmonization of data and meaningful comparisons with equivalent datasets. Additionally, we collect or calculate health indicators for the routine monthly aggregate reports required by the MOUs (Table 1).

Externally-funded PER data clerks are embedded at the facilities and project-augmented data collection is accommodated within the routine patient and document flow without disruption of clinical care.

For peer review only

The provincial government of the WC operates as a single provider of public sector health services. A 9-digit numeric folder number which is common across the health platform for a given patient facilitates the harmonization of all electronic health records within the Provincial Health Data Centre (PHDC), the information exchange that consolidates all electronic administrative, pharmacy, laboratory, and disease-specific information[14]. PER data are recorded against this identifier and contribute to the PHDC.

All MOUs use the Primary Health Care Information Service (PHCIS) electronic medical records system which records attendance against patient identifiers, and ART in WLHIV. PHCIS automatically generates a unique folder number for live infants at birth, providing electronic linkage between mother and baby. Clinicom performs this function at all hospitals. Data are imported daily by the PHDC[14].

Completeness of Medicine Exposure data

Electronic dispensing data in the PHDC is augmented by the PER which captures medicine exposures elicited from the women during the clinical consultation and ward-stock medicines recorded by clinicians in the MCR. The PER also records some lifestyle factors (weight gain, alcohol, tobacco, recreational drugs) that may act as confounders for certain outcomes. Combining the electronic pharmacy data in the PHDC strengthens the ascertainment of exposures, providing a complete list of medication dispensed from public sector pharmacies. Using multiple data sources for this has been shown to provide a more complete picture of antenatal medicine use essential for pregnancy exposure research[3, 22, 23].

For peer review only

Outcome Ascertainment

Information on neonatal outcomes such as vital status, birth weight, gestational age, and APGAR scores tend to be consistently captured across the cohort. The key findings of the neonatal surface examinations, although often perfunctory, usually result in the recording of notable physical anomalies. Internal anomalies such as cleft palate, hip dysplasias, and cardiac anomalies as well as more subtle dysmorphic features may be missed at the time of the initial neonatal examination. Details of neonatal deaths, and major congenital disorders often require review of inpatient records at the delivery facilities.

PER data are imported daily into the PHDC and linked using patient identifiers, providing a comprehensive electronic clinical record at the level of the individual which is accessible to the attending clinicians. Both systems benefit greatly from this design. The PER allows for validation of the provincial dataset as relates to pregnancy and delivery, and the PHDC is able to identify missing outcomes (often at sites outside the referral axes) or exposures (from electronic pharmacy dispensing) not included in the PER.

<u>Findings to date</u>

Between 01 September 2016 and 31 August 2021, 31 346 pregnancies were recorded in the PER. To assess robustness of the dataset, we analysed data for a subset of women who attended their first visit to antenatal care between 01 January 2018 and 31 December 2019 (Table 2). Over this two-year period, 14 527 individual pregnancies were recorded in the PER: 9435 and 5092 at the urban and rural site, respectively. Outcomes were determined for 93.4% of pregnancies (n = 13 574). Gestational dating scans were performed in 38.5% (n = 5583) of all enrolees, of whom 60% (n = 3345) were ≤22 weeks, facilitating more precise gestational dating at birth as well as timing of exposures.

For peer review only

Where relevant, we compared rates of key adverse birth outcomes in the PER with official aggregate routine indicator data for the WC[17, 24-26], derived from register aggregates reported through the District Health Information System (Table 3).

Published and other outputs

We conducted an initial baseline assessment comparing clinical records to dispensing data before the implementation of the PER[15] and recently updated the analysis demonstrating the value of combining PER and electronic pharmacy data in improving medicine exposure ascertainment[3]. We are currently investigating the impact of data source on gestational age (Malaba T, manuscript in preparation) and hypertensive disorders of pregnancy[27]. PER data have contributed to population-based analyses describing the use and safety of sodium valproate and isoniazid for TB preventive therapy in pregnancy[28, 29]. In addition, initiation of the PER provided the opportunity to host a workshop, *Building Teratovigilance Capacity in Africa,* which provided networking and training opportunities to 60 delegates from sub-Saharan Africa https://globalpharmacovigilance.tghn.org/resources/building-teratovigilance-capacity-africa/.

For peer review only

Strengths and Weaknesses

Strengths

The integration of the PER within the PHDC greatly increases the completeness of the data. It facilitates identification of pregnancy outcomes at facilities outside our sentinel referral chains reducing loss to follow-up. Harmonization and triangulation of two data sources for medicine exposures (i.e., clinical records and electronic pharmacy records) provides a more robust summary of

exposures than either alone[3, 10, 15]. These systems comprise unique infrastructure able to address clinical and public health concerns in a low-middle income setting.

Accurate timing of exposures over the course of pregnancy is crucial to assess potential associations with adverse pregnancy outcomes. Collecting multiple reference points for gestational age (i.e., neonatal record, ultrasound, last menstrual period, symphysis-fundal height) enabled the development of a hierarchy of methods and the allocation of a confidence score to the reported gestational age[30-32]. This offers an advantage over insurance claims datasets which are often used to determine safety information and in which pregnancy and gestational age must be inferred from clinical coding alone.

In line with WHO recommendations[13], all women attending the PER primary care sites are enrolled and we reflect background rates of important pregnancy parameters similar to what is expected from national aggregate data. This will be expanded to include background rates for congenital disorders, data which are lacking in South Africa[33]. This structure also allows for the analysis of multiple current and potential future exposures and emerging health concerns e.g., novel medicines and vaccines such as for SARS-CoV-2.

From the outset, it was important to avoid a parallel system and support project sustainability. The PER has been integrated into the existing clinical and clerical routines and uses local electronic health information platforms. It allows for electronic generation of key monthly indicators at primary care sites that are otherwise collected by hand.

For peer review only

As the cohort expands, capacity to conduct nested studies that facilitate signal detection and signal verification of potential or suspected teratogens will improve. The collection of individual-level data in a large prospectively enrolled cohort, representative of both urban and rural WC populations who use public sector services will support more robust analyses that can better account for confounding factors in such observational data.

Weaknesses

The PER digitizes clinical data recorded in operational stationery and we cannot control for data quality nor account for missing data in the source documents. To address this, we have engaged in on-going training at the sites with an emphasis on drug history taking, medical record-keeping and neonatal examination offering in-person teaching and video tutorials. Clinical staff have been provided with Medicine Identification Aids with photographs of common formulations and packaging, and the WHO Birth Defects atlas[34]. However, misclassification remains a potential risk.

Notwithstanding the advantages of the individual-level data available within the PHDC, data are limited to those that are entered into one of the electronic medical records systems used in the public sector. In terms of medicine exposures, the PER documents dispensed medication which may not reflect actual use. In addition, medicines obtained outside of the public sector systems, from private doctors or over-the-counter from pharmacies are not included unless they are noted in the clinical records[3]. Similarly, traditional and complementary medicines lack a linked electronic footprint and are not included.

The PER database is parsimonious by design and necessity and we are unable to account for unmeasured confounders. However, data fields are collected for the entire cohort who are all drawn

from the same geographical areas served by the primary care clinics. Additionally, we record limited data on lifestyle factors relevant in pregnancy (weight gain, exposure to tobacco, alcohol, recreational drugs) which are lacking from equivalent population datasets based on insurance claims data.

Collaboration

As with the PHDC within which it is located, the PER can address clinical, operational and research needs, and data access is specific to each. Aggregate reports are available to managers. Data are anonymised using standard protocols for de-identifying records before they are shared with researchers who are not directly engaged in the women's clinical care. It is anticipated that such de-identified individual-level data may be shared as part of the South African National Pregnancy Registry[35] and with similar PER initiatives regionally or internationally[36]. Data-sharing commitments are particularly relevant to research of rare events such as congenital disorders[13]. The PHDC has in-built privacy systems and strict governance structures managing the protection and use of health data for both service and research purposes and these apply to the PER[14].

Patient and public involvement

The PER is integrated into the data collection and curation services of the Western Cape Government Department of Health and clinical and other service providers have engaged with the project since its inception. The data are available to managers as aggregate reports and to contribute to the electronic clinical records accessible by clinicians. Feedback from users contributes iteratively to optimization of the PER to improve health outcomes for pregnant women and infants.

For peer review only

Conclusions

Research on medicine safety in pregnancy requires data on individual pregnancies, mother-infant linkage, medication exposure, gestational age at exposure, and maternal and birth outcomes. Data completeness and robustness continues to improve with on-going training, evolution of routine clinical information systems, and increasing political focus on pregnancy exposures. The cohort is well-placed to detect large signals in pregnancy outcomes as novel maternal exposures are introduced, and to contribute to cohort harmonization for rarer outcomes and address the lack of information on congenital disorders in Africa.

Ethics approval

The WC PER has been approved by the Faculty of Health Sciences Human Research Ethics Committees of the University of Cape Town (HREC: 749/2015) and Stellenbosch University (N17/04/040, N20/08/084), and the Western Cape Government Health Research Committee.

Funding

This work was supported by the US National Institutes of Health [R01HD080465, U01AI069911, U01AI069924, K43TW010683], Bill and Melinda Gates Foundation [INV-004508; 1164272; 1191327; INV-004657], the Wellcome Trust [203135/Z/16/Z], the International AIDS Society [2017-518/SLO], the United States Agency for International Development [72067418CA00023] and the US Centers for Disease Control and Prevention [GH001934].

For peer review only

Acknowledgements

We acknowledge and thank the PER data teams in Cape Town and Worcester, and the clinical and management staff at the facilities. The support and investment of the subdistrict and provincial health management is crucial to maintaining the initiative. We further acknowledge colleagues from the Western Cape Government: Health and the City of Cape Town.

Author contributions

Conception and design: EK, UM, AB, ALS, MAD, LM

Design and Implementation including data systems: EK, UM, ALS, GP, KF, JE

Data harmonization: AH, FP, JE, AB, MAD, EK

Clinical oversight: GP, MK, CS, NR, SG, AO, KF

Data cleaning & analysis: EK, ALS, UM, KA, AH

All authors critically reviewed the manuscript.

Conflict of interests

EK, AB, MAD and KA received funding from Viiv Healthcare unrelated to this project.

The authors declare no conflicts of interest.

For peer review only

References

1. Daw JR, Hanley GE, Greyson DL, Morgan SG. Prescription drug use during pregnancy in developed countries: a systematic review. Pharmacoepidemiol Drug Saf. 2011;20(9):895-902.
2. Mohseni M, Azami-Aghdash S, Gareh Sheyklo S, Moosavi A, Nakhaee M, Pournaghi-Azar F, et al. Prevalence and Reasons of Self-Medication in Pregnant Women: A Systematic Review and Meta-Analysis. Int J Community Based Nurs Midwifery. 2018;6(4):272-84.
3. van der Hoven J, Allen E, de Waal R, Cois A, Maartens G, Myer L, et al. Antenatal medicine use in South African women: a comparison of three methods of ascertainment. 2021.
4. Zash R, Holmes L, Diseko M, Jacobson DL, Brummel S, Mayondi G, et al. Neural-Tube Defects and Antiretroviral Treatment Regimens in Botswana. N Engl J Med. 2019;381(9):827-40.
5. Zash R, Makhema J, Shapiro RL. Neural-Tube Defects with Dolutegravir Treatment from the Time of Conception. N Engl J Med. 2018;379(10):979-81.
6. Gupta A, Montepiedra G, Aaron L, Theron G, McCarthy K, Bradford S, et al. Isoniazid Preventive Therapy in HIV-Infected Pregnant and Postpartum Women. N Engl J Med. 2019;381(14):1333-46.
7. Bianchi DW, Kaeser L, Cernich AN. Involving Pregnant Individuals in Clinical Research on COVID-19 Vaccines. JAMA. 2021;325(11):1041-2.
8. Holmes LB, Wyszynski DF. North American antiepileptic drug pregnancy registry. Epilepsia. 2004;45(11):1465.

For peer review only

9. Scheuerle A, Covington D. Clinical review procedures for the Antiretroviral Pregnancy Registry. Pharmacoepidemiol Drug Saf. 2004;13(8):529-36.
10. Gelperin K, Hammad H, Leishear K, Bird ST, Taylor L, Hampp C, et al. A systematic review of pregnancy exposure registries: examination of protocol-specified pregnancy outcomes, target sample size, and comparator selection. Pharmacoepidemiol Drug Saf. 2017;26(2):208-14.
11. Berrueta M, Ciapponi A, Bardach A, Cairoli FR, Castellano FJ, Xiong X, et al. Maternal and neonatal data collection systems in low- and middle-income countries for maternal vaccines active safety surveillance systems: A scoping review. BMC Pregnancy Childbirth. 2021;21(1):217.
12. Mehta U, Kalk E, Fairlie L, Boulle A, Rees H. Why South Africa urgently needs to support the development of pregnancy exposure registries. S Afr Med J. 2019;109(5):294-5.
13. Mehta U, Clerk C, Allen E, Yore M, Sevene E, Singlovic J, et al. Protocol for a drugs exposure pregnancy registry for implementation in resource-limited settings. BMC Pregnancy Childbirth. 2012;12:89.
14. Boulle A, Heekes A, Tiffin N, Smith M, Mutemaringa T, Zinyakatira N, et al. Data Centre Profile: The Provincial Health Data Centre of the Western Cape Province, South Africa. Int J Popul Data Sci. 2019;4(2):1143.
15. Mehta U, Heekes A, Kalk E, Boulle A. Assessing the value of Western Cape Provincial Government health administrative data and electronic pharmacy records in ascertaining medicine use during pregnancy. S Afr Med J. 2018;108(5):439-43.
16. Okoli GN, Myles P, Murray-Thomas T, Shepherd H, Wong ICK, Edwards D. Use of Primary Care Data in Research and Pharmacovigilance: Eight Scenarios Where Prescription Data are Absent. Drug Saf. 2021.
17. Day C, Gray C, Padayachee T, Cois A. Chapter 20, Health and Related Indicators. South African Health Review 2020. 2020.
18. South African National Department of Health. Guideline for the Prevention of Mother to Child Transmission of Communicable Infections. Pretoria. November 2019.
19. World Health Organization. WHO recommendations on antenatal care for a positive pregnancy experience. Geneva: WHO Library Cataloguing-in-Publication Data; 2016.
20. Holmes LB, Nasri HZ, Hunt AT, Zash R, Shapiro RL. Limited surface examination to evaluate potential teratogens in a resource-limited setting. Birth Defects Res. 2021;113(9):702-7.
21. Bonhoeffer J, Kochhar S, Hirschfeld S, Heath PT, Jones CE, Bauwens J, et al. Global alignment of immunization safety assessment in pregnancy - The GAIA project. Vaccine. 2016;34(49):5993-7.
22. Andrade SE, Berard A, Nordeng HME, Wood ME, van Gelder M, Toh S. Administrative Claims Data Versus Augmented Pregnancy Data for the Study of Pharmaceutical Treatments in Pregnancy. Curr Epidemiol Rep. 2017;4(2):106-16.
23. Grzeskowiak LE, Gilbert AL, Morrison JL. Investigating outcomes associated with medication use during pregnancy: a review of methodological challenges and observational study designs. Reprod Toxicol. 2012;33(3):280-9.
24. Massyn N, Day C, Ndlovu N, Padayachee T. District Health Barometer 2019/2020. Durban: Health Systems Trust; 2020.
25. National Perinatal Mortality and Morbidity Committee. NAPEMMCO Trennial Report 2017 - 2019. 2017-2019.
26. Provincial Perinatal Morbidity and Mortality Commitee. Western Cape Saving Babies Trennial Report 2017 -2019. 2020.
27. Slogrove A, Davies MA, Williams P, Abrams E, Theron G, Cotton M, et al., editors. Hypertensive Disorders in Pregnancy and HIV: A Province-wide Cohort Analysis during 2018 and 2019 in the Western Cape, South Africa. 13th International Workshop on HIV Pediatrics; 2021 16-17 July 2021; virtual.
28. Kalk E, Heekes A, Mehta U, de Waal R, Jacob N, Cohen K, et al. Safety and Effectiveness of Isoniazid Preventive Therapy in Pregnant Women Living with Human Immunodeficiency Virus on Antiretroviral Therapy: An Observational Study Using Linked Population Data. Clin Infect Dis. 2020;71(8):e351-e8.

For peer review only

29. Mehta U, Smith M, Kalk E, Hayes H, Swart A, Tucker L, et al. Understanding and Responding to Prescribing Patterns of Sodium Valproate-Containing Medicines in Pregnant Women and Women of Childbearing Age in Western Cape, South Africa. Drug Saf. 2021;44(1):41-51.
30. Geerts L, Poggenpoel E, Theron G. A comparison of pregnancy dating methods commonly used in South Africa: a prospective study. S Afr Med J. 2013;103(8):552-6.
31. Malaba TR, Newell ML, Madlala H, Perez A, Gray C, Myer L. Methods of gestational age assessment influence the observed association between antiretroviral therapy exposure, preterm delivery, and small-for-gestational age infants: a prospective study in Cape Town, South Africa. Ann Epidemiol. 2018;28(12):893-900.
32. Quinn JA, Munoz FM, Gonik B, Frau L, Cutland C, Mallett-Moore T, et al. Preterm birth: Case definition & guidelines for data collection, analysis, and presentation of immunisation safety data. Vaccine. 2016;34(49):6047-56.
33. Lebese V, Aldous C, Malherbe HL. South African congenital disorders data, 2006 - 2014. S Afr Med J. 2016;106(10):992-5.
34. WHO/CDC/ICBDSR. Birth defects surveillance: atlas of selected congenital anomalies. Geneva: World Health Organization; 2014.
35. Buhle. U. Understanding Birth Outcomes for Mothers and Infants: Building Healthcare by Linking Exposures 2021 [Available from: https://www.ubomibuhle.org.za/.
36. IeDEA-EA. Measuring Adverse Pregnancy & Newborn Outcomes (MANGO): an IedEA Collaboration Study 2021 [Available from: https://globalhealth.iu.edu/research/iedea-main-page/]

For peer review only

Table 1. Summary of Data Elements in the PER

**Variables collected**	**Calculated parameters**	**MOU aggregate statistics**
Antenatal<br>Maternal date of birth<br>Date of first antenatal visit<br>Last menstrual period<br>Parity, Gravidity<br>Obstetric & Medical history<br>Chronic medication<br>Height, mid-upper arm circumference, weight, blood pressure, urinalysis<br>Symphysis fundal height<br>Alcohol, tobacco, drug use<br>Number of antenatal visits	Gestational age at first antenatal visit	Number of first visits<br>Number of women first ANC < 20 weeks<br>Number age < 20 years or > 38 years<br>Number grand multipara (≥ 5 deliveries)<br>Number high blood pressure/proteinuria
Vertical transmission of HIV<br>HIV status at first antenatal visit<br>Subsequent positive HIV test<br>HIV treatment incl. regimen switches<br>CD4 count<br>Viral load<br>HIV-exposed infant HIV-PCR	Number of women at high risk of vertical HIV transmission [18]<br>ART in hand at estimated time of conception<br>ART in hand at delivery	Number of women living with HIV:<br>Before pregnancy<br>During pregnancy<br>Number of women on ART (1st & 2nd line):<br>Before, during pregnancy<br>VL unsuppressed at pregnancy & delivery<br>Number of infant birth HIV-PCR
Ultrasound<br>Gestational age<br>Abnormalities<br>Expected date of delivery		Number of ultrasounds conducted<br>Number multiple pregnancies
Maternal outcome<br>Facility-based death	Vital status	Maternal death
Peri-partum<br>Date & site of outcome		Number of deliveries

For peer review only

Method of delivery<br>Gestational age at outcome	Prematurity (< 37 completed weeks gestation)	
Pregnancy outcome<br>Livebirth<br>Stillbirth<br>Miscarriage<br>Termination of pregnancy<br>Ectopic pregnancy<br>Molar pregnancy	Gestational age at pregnancy outcome	Number of livebirths, stillbirths, miscarriages
Neonate<br>Date of birth<br>Sex, APGAR scores<br>Gestational age<br>Birth weight, length, head circumference, foot length<br>Neonatal surface examination<br>Abnormalities noted	Gestational age at birth<br>Low birth weight (< 2500g)<br>Prematurity (< 37 completed weeks gestation)<br>Neonatal death	Number of low birth weight infants<br>Number premature infants<br>Number neonatal deaths<br>Perinatal mortality rate

ANC – antenatal care; ART – antiretroviral therapy; HIV – Human Immune Deficiency Virus; PCR – polymerase chain reaction

For peer review only

Table 2. Maternal and obstetric characteristics of the cohort 2018-2019

Variable	PER total n = 14 527	PER urban n = 9435 (65%)	PER rural n = 5092 (35%)
Age (years) median (IQR)	27 (23 – 32)	28 (23 – 33)	26 (22 – 31)
Living with HIV at pregnancy outcome	3931 (27.1)	3241 (34.3)	690 (13.6)
Obstetric ultrasound present n (%)	5583 (38.4)	4063 (43.1)	1520 (29.9)
Early ultrasound (i.e. < 22 weeks) n (% of US)	3345 (59.9)	2393 (58.9)	952 (62.6)
Gestational age at birth (weeks) median (IQR)	40 (37 – 40)	40 (36 – 40)	39 (35 – 40)
Birth weight (grams) median (IQR)	3100 (2750 – 3440)	3140 (2800 – 3480)	2975 (2575 – 3320)
Low birth weight[a] n (%)	1736 (12.0)	879 (9.3)	857 (16.8)
Premature birth[b] n(%)	2949 (20.3)	1735 (18.4)	1214 (23.8)
Pregnancy outcome n (%)			
Live birth	12 419 (85.5)	1189 (82.3)	4630 (90.9)
Still birth	296 (2.0)	180 (1.9)	116 (2.3)
Neonatal death[c]	109 (0.8)	71 (0.5)	36 (0.7)
Miscarriage	395 (2.7)	318 (3.4)	77 (1.5)
Ectopic pregnancy	82 (0.6)	60 (0.6)	22 (0.4)
Termination of pregnancy	273 (1.9)	223 (2.4)	50 (1.0)
Unknown	953 (6.6)	792 (8.4)	161 (3.1)
Delivery method[d] n(%)			
Born before arrival at birthing facility	608 (4.7)	245 (3.1)	363 (7.6)
Vaginal delivery	7587 (59.2)	4655 (57.9)	2932 (61.3)
Assisted delivery[e]	140 (1.1)	51 (0.6)	89 (1.9)
Caesarean section	3416 (26.6)	2411 (30.0)	1005 (21.0)
Unknown	1073 (8.4)	680 (8.5)	393 (8.2)
Infant outcome[d] n(%)			
Stillborn	296 (2.3)	180 (2.2)	116 (2.4)
Early neonatal death[c]	80 (0.6)	55 (0.7)	25 (0.5)
Late neonatal death	29 (0.2)	18 (0.2)	11 (0.2)
Alive	12 419 (96.8)	7798 (96.9)	4630 (96.8)
Tobacco use[f] n(%)			
Current user	1297 (8.9)	87 (0.9)	1210 (23.8)
Past user	55 (0.4)	13 (0.1)	42 (0.8)
Never user	9997 (68.8)	7222 (76.5)	2775 (54.5)
Not reported	3178 (21.9)	2113 (14.5)	1065 (7.3)
Alcohol use[f] n(%)			
Current user	588 (4.1)	339 (3.6)	249 (4.9)
Past user	167 (1.2)	66 (0.7)	101 (2.0)
Never user	10 570 (72/8)	6885 (73.0)	3685 (72.4)
Not reported	3202 (22.0)	2145 (14.8)	1057 (7.3)

[a] birthweight<2500g; liveborn infants only

[b] birth < 37 completed weeks gestation; liveborn infants only

For peer review only

For peer review only - http://bmjopen.bmj.com/site/about/guidelines.xhtml

[c] neonatal death: death before 28 days of life; early neonatal death: death before 7days of life; late neonatal death: death between 8 and 28 days of life

[d] viable pregnancies (i.e >27 weeks gestation(17)) (n=12 824)

[e] forceps or vacuum delivery

[f] reported at first antenatal visit

For peer review only

Table 3. Comparison between PER reported or calculated PER outcomes and aggregate indicators in formal provincial information systems

Indicator	PER total n = 14 527	PER urban n = 9435 (65%)	PER rural n = 5092 (35%)	Reported aggregate for the Western Cape 2017 - 2019[a]
Still birth[b] n (%)<br><br>Per 1000 births	296 (2.0)<br><br>20.0	180 (1.9)<br><br>19.1	116 (2.4)<br><br>24.0	2.2%[26]<br><br>18.5[17]<br>22.1[25,26]
Neonatal death in facility rate[c] per 1000 live births	8.7	9.2	7.7	8.9[17,25]
Perinatal mortality rate[d] per 1000 births	29	29	29	25.6[17]<br>27.9[25]<br>29.1[26]
Low birth weight[e] n(%)	1737 (12.0)	879 (9.3)	857 (16.8)	14.9% urban subdistrict<br>18.4% rural subdistrict[26]
Maternal mortality in facility ratio per 100 000 live births		63.5	Insufficient data	43.6 – 66.8[25]
Teenage pregnancies (10 – 19 years) n(%)	929 (6.4)	450 (4.8)	497 (9.4)	3.5% urban subdistrict<br>7.3% rural subdistrict[26]
Caesarean section rate per 1000 births	3416 (26.6)	2411 (30.0)	1005 (21.0)	28.9[25] - 29.3[26]

[a] includes aggregate reports compiled from the District Health Information System and Perinatal Problem Identification Programme[17, 25-26]

[b] delivery of a baby with no signs of life after 27 completed weeks of gestation (i.e., viable baby born dead)

[c] death before 28 days of life

[d] still birth plus neonatal deaths <8 days per 1000 births

[e] birthweight<2500g; liveborn infants only

For peer review only - http://bmjopen.bmj.com/site/about/guidelines.xhtml

BMJ Open

Cohort Profile: The Western Cape Pregnancy Exposure Registry (WCPER)

Journal:	*BMJ Open*
Manuscript ID	bmjopen-2021-060205.R1
Article Type:	Cohort profile
Date Submitted by the Author:	23-Mar-2022

Complete List of Authors:	Kalk, Emma; University of Cape Town Faculty of Health Sciences, Centre for Infectious Disease Epidemiology & Research<br>Heekes, Alexa; University of Cape Town Faculty of Health Sciences, Centre for Infectious Disease Epidemiology & Research; Western Cape Department of Health, Health Intelligence Directorate<br>Slogrove, Amy; Stellenbosch University, Ukwanda Centre for Rural Health, Department of Global Health; Stellenbosch University, Department of Paediatrics & Child Health<br>Phelanyane, Florence; University of Cape Town Faculty of Health Sciences, Centre for Infectious Disease Epidemiology & Research; Western Cape Department of Health, Health Intelligence Directorate<br>Davies, Mary-Ann; University of Cape Town Faculty of Health Sciences, Centre for Infectious Disease Epidemiology & Research; Western Cape Department of Health, Health Intelligence Directorate<br>Myer, Landon; University of Cape Town Faculty of Health Sciences, Division of Epidemiology & Biostatistics<br>Euvrard, Jonathan; University of Cape Town Faculty of Health Sciences, Centre for Infectious Disease Epidemiology & Research; Western Cape Department of Health, Health Intelligence Directorate<br>Kroon, Max; University of Cape Town Faculty of Health Sciences, Neonatology; Mowbray Maternity Hospital, Neonatal Services<br>Petro, Greg; University of Cape Town Faculty of Health Sciences, Department of Obstetrics & Gynaecology; New Somerset Hospital, Maternity Services<br>Fieggen, Karen; University of Cape Town Faculty of Health Sciences, Division of Human Genetics; Groote Schuur Hospital, Medical Genetics Services<br>Stewart, Chantal; University of Cape Town Faculty of Health Sciences, Department of Obstetrics & Gynaecology; Mowbray Maternity Hospital, Maternity Services<br>Rhoda, Natasha; University of Cape Town Faculty of Health Sciences, Department of Paediatrics and Child Health; Mowbray Maternity Hospital, Neonatal Services<br>Gebhardt, Stefan; Stellenbosch University, Department of Obstetrics & Gynaecology; Tygerberg Hospital, Maternity Services<br>Osman, Ayesha; University of Cape Town Faculty of Health Sciences, Department of Obstetrics & Gynaecology; Groote Schuur Hospital, Maternity Services<br>Anderson, Kim; University of Cape Town Faculty of Health Sciences, Centre for Infectious Disease Epidemiology & Research<br>Boulle, Andrew; University of Cape Town Faculty of Health Sciences,

	Centre for Infectious Disease Epidemiology & Research; Western Cape Department of Health, Health Intelligence Directorate<br>Mehta, Ushma; University of Cape Town Faculty of Health Sciences, Centre for Infectious Disease Epidemiology & Research
**Primary Subject Heading**:	Public health
Secondary Subject Heading:	Epidemiology, Health services research, Obstetrics and gynaecology, Pharmacology and therapeutics, Public health
Keywords:	PUBLIC HEALTH, EPIDEMIOLOGY, Maternal medicine < OBSTETRICS, Fetal medicine < OBSTETRICS

SCHOLARONE™
Manuscripts

I, the Submitting Author has the right to grant and does grant on behalf of all authors of the Work (as defined in the below author licence), an exclusive licence and/or a non-exclusive licence for contributions from authors who are: i) UK Crown employees; ii) where BMJ has agreed a CC-BY licence shall apply, and/or iii) in accordance with the terms applicable for US Federal Government officers or employees acting as part of their official duties; on a worldwide, perpetual, irrevocable, royalty-free basis to BMJ Publishing Group Ltd ("BMJ") its licensees and where the relevant Journal is co-owned by BMJ to the co-owners of the Journal, to publish the Work in this journal and any other BMJ products and to exploit all rights, as set out in our licence.

The Submitting Author accepts and understands that any supply made under these terms is made by BMJ to the Submitting Author unless you are acting as an employee on behalf of your employer or a postgraduate student of an affiliated institution which is paying any applicable article publishing charge ("APC") for Open Access articles. Where the Submitting Author wishes to make the Work available on an Open Access basis (and intends to pay the relevant APC), the terms of reuse of such Open Access shall be governed by a Creative Commons licence – details of these licences and which Creative Commons licence will apply to this Work are set out in our licence referred to above.

Other than as permitted in any relevant BMJ Author's Self Archiving Policies, I confirm this Work has not been accepted for publication elsewhere, is not being considered for publication elsewhere and does not duplicate material already published. I confirm all authors consent to publication of this Work and authorise the granting of this licence.

For peer review only

Cohort Profile: The Western Cape Pregnancy Exposure Registry (WCPER)

Authors: Emma Kalk[1*], Alexa Heekes[1,2,] Amy Slogrove[3,4], Florence Phelanyane[1,2], Mary-Ann Davies[1,2], Landon Myer[5], Jonathan Euvrard[1,2], Max Kroon[6,7], Greg Petro[8,9], Karen Fieggen[10,11], Chantal Stewart[8,12], Natasha Rhoda[6,7], Stefan Gebhardt[13,14], Ayesha Osman[8,15], Kim Anderson[1], Andrew Boulle[1,2†], Ushma Mehta[1†]

1. Centre for Infectious Disease Epidemiology & Research and Division of Public Health Medicine, School of Public Health & Family Medicine, University of Cape Town, South Africa

2. Health Intelligence Directorate, Western Cape Government Health, Cape Town, South Africa

3. Department of Paediatrics & Child Health, Faculty of Medicine & Health Sciences, Stellenbosch University, Worcester, South Africa

4. Ukwanda Centre for Rural Health, Department of Global Health, Faculty of Medicine & Health Sciences, Stellenbosch University, Worcester, South Africa

5. Division of Epidemiology & Biostatistics, School of Public Health & Family Medicine, University of Cape Town, South Africa

6. Department of Paediatrics and Child Health, University of Cape Town, South Africa

7. Mowbray Maternity Hospital, Neonatal Services, Cape Town, South Africa

8. Department of Obstetrics & Gynaecology, University of Cape Town, South Africa

9. New Somerset Hospital, Maternity Services, Cape Town, South Africa

10. Division of Human Genetics, University of Cape Town, Cape Town, South Africa

11. Groote Schuur Hospital, Medical Genetics Service, Cape Town, South Africa

12. Mowbray Maternity Hospital, Maternity Services, Cape Town, South Africa

13. Department of Obstetrics & Gynaecology, Stellenbosch University, Cape Town, South Africa

For peer review only - http://bmjopen.bmj.com/site/about/guidelines.xhtml

14. Tygerberg Hospital Maternity Services, Cape Town, South Africa

15. Groote Schuur Hospital, Maternity Services, Cape Town South Africa

*corresponding author: Centre for Infectious Disease Epidemiology & Research, School of Public Health & Family Medicine, 5th floor Falmouth Building, University of Cape Town Faculty of Health Sciences, Anzio Road, Observatory, Cape Town

emma.kalk@uct.ac.za

† Joint senior authors

For peer review only

For peer review only - http://bmjopen.bmj.com/site/about/guidelines.xhtml

ABSTRACT

Purpose: The Western Cape Pregnancy Exposure Registry (PER) was established at two public sector healthcare sentinel sites in the Western Cape province, South Africa to provide on-going surveillance of drug exposures in pregnancy and associations with pregnancy outcomes.

Participants: Established in 2016, all women attending their first antenatal visit at primary care obstetric facilities were enrolled and followed to pregnancy outcome regardless of the site (i.e., primary, secondary, tertiary facility). Routine operational obstetric and medical data are digitized from the clinical stationery at the health care facilities. Data collection has been integrated into existing services and information platforms and supports routine operations. The PER is situated within the Provincial Health Data Centre, an information exchange that harmonizes and consolidates all health-related electronic data in the province. Data are contributed via linkage across a unique identifier. This relationship limits the missing data in the PER, allows validation and avoids misclassification in the population-level dataset.

Findings to date: Approximately 5000 and 3500 pregnant women enter the dataset annually at the urban and rural sites, respectively. As of August 2021, >30 000 pregnancies have been recorded and outcomes have been determined for 93%. Analysis of key obstetric and neonatal health indicators derived from the PER are consistent with the aggregate data in the District Health Information System.

Future plans: This represents significant infrastructure, able to address clinical and epidemiological concerns in a low/middle-income setting.

Key words

Pregnancy Exposure Registry, Pharmacovigilance, Surveillance

For peer review only

Strengths and Limitations of this Study

The Western Cape Pregnancy Exposure Registry (PER) provides on-going surveillance of drug exposures in pregnancy and associations with pregnancy outcomes.

Data collection is integrated into existing services and information platforms and supports routine operations.

The PER is situated within the Provincial Health Data Centre, an information exchange that harmonizes and consolidates all health-related electronic data which limits missing data, allows validation and avoids misclassification in the population-level dataset.

The PER digitizes clinical data recorded in operational stationery and we cannot control for data quality nor account for missing data in the source documents nor for unmeasured confounders.

Medicines obtained outside the public sector systems and traditional and complementary medicines are not included unless they are documented in the clinical stationery.

For peer review only

Cohort Profile: The Western Cape Pregnancy Exposure Registry

Introduction

Assessing medicine and vaccine safety in pregnancy requires on-going surveillance across multiple settings. In high-income countries, reviews of outpatient prescriptions and self-medication during pregnancy estimated exposure rates of up to 93% and 43%, respectively, excluding vitamins and supplements[1, 2]. Reports from Africa, the site of mass prevention and treatment campaigns for HIV, tuberculosis and malaria, are less frequent: we estimate that 79% - 99% of women in Cape Town use medicines antenatally[3].

Pregnant women have been systematically excluded from pharmaceutical trials and the efficacy, dosing and safety of many medicines used during pregnancy are uncertain or findings delayed until after the product is licensed and in use. Post-authorization safety assessments have traditionally relied on passive reporting of suspected medicine-related adverse events. Such systems have been limited by their dependence on voluntary reporting, variable data quality, absence of background rates of adverse birth outcomes including common congenital disorders, and lack of data to establish a denominator.

Recently, pharmacovigilance in pregnancy has drawn public and political attention following concerns about the initial association observed between the antiretroviral integrase inhibitor, dolutegavir, and neural tube defects[4, 5], the potential risk of isoniazid preventive therapy in women living with HIV[6] (WLHIV), and SARS-CoV-2 vaccines[7]. With all these exposures, synthesis and meta-analysis of the available data has been re-assuring and the World Health Organization (WHO) guidelines report no contra-indication to their use in pregnant and breast-feeding women [8-10]. In addition, there have

For peer review only - http://bmjopen.bmj.com/site/about/guidelines.xhtml

been increased calls globally for the inclusion of pregnant women in clinical trials for new therapeutic and preventive agents, particularly in the field of infectious disease[11-14].

Pregnancy Exposure Registries (PER) are a form of surveillance, designed to iteratively detect adverse events within a defined pregnant population. Importantly, the prospective nature of PER allows collection of exposure and other data before the pregnancy outcome is known. The pharmaceutical industry maintains drug-specific registries for medicines and/or drug classes with known/suspected teratogenic effects or as part of post-marketing commitments; e.g., the Antiretroviral Pregnancy and Anti-Epileptic Drug Registries[15, 16]. In addition, teratology information services may collect data on pregnancy exposures. These PER depend on voluntary enrolment by clinicians and/or women, and many do not directly collect data from comparator groups but rely either on internal comparators or on an identified external comparator to provide background prevalence data[17]. Background rates of adverse maternal and obstetric outcomes are necessary to determine deviations from expected proportions (signals). Such data may be limited or lacking in low- and middle-income countries[18, 19] or differ sufficiently from the source population so as to introduce bias (e.g., use of the Metropolitan Atlanta Congenital Defects Program as external comparator for USA-based studies[17].)

The UNICEF/UNDP/World Bank/WHO Special Programme for Research & Training in Tropical Diseases (TDR) has developed a PER approach for resource-limited settings aimed at prospective data collection on exposures in a cohort of pregnant women attending antenatal care services at sentinel sites. Important for validity and causality determination, the approach recommends inclusion of *all* women presenting to the site to allow concurrent establishment of background rates and assessment of multiple potential exposures[20].

For peer review only - http://bmjopen.bmj.com/site/about/guidelines.xhtml

The Western Cape (WC) PER was established in Cape Town in 2016, adapted from the TDR template. It was nested within the province-wide health information exchange, a component within a larger project designed to assess the impact of WHO Option B+ for vertical HIV transmission prevention (i.e., universal lifelong antiretroviral therapy [ART] for pregnant and breast-feeding women) at the population and individual levels[21]. Situating the PER within the linked information exchange avoided some of the limitations of exclusive primary-care databases in that both electronic inpatient and outpatient prescriptions are recorded as well as those from specialist and other off-site clinics, sources which may be absent from primary-care records[3, 22, 23]. The design also supports augmentation of the electronic clinical record for enrolled women, while providing a more secure, sustainable, and ethically-viable platform for capturing clinical data on mothers and infants.

We took a pragmatic approach to the establishment of the PER based on the availability of resources and the desire to integrate into existing systems and operational routines, avoiding a parallel infrastructure and supporting longevity. Data generated by the initiative are available for the evaluation and improvement of clinical care as well as epidemiological review.

<u>Cohort description</u>

The PER has been established at two sentinel sites in the WC. Gugulethu Midwife Obstetric Unit (GMOU) provides obstetric care to approximately 5000 women annually in Gugulethu, Cape Town a low-income area with high unemployment and an antenatal HIV prevalence of approximately 30%. GMOU refers patients to Mowbray Maternity (secondary) and Groote Schuur (tertiary) Hospitals. About half of all women who attend GMOU are referred to hospital, antenatally or perinatally. Worcester MOU (WMOU) is situated adjacent to the Worcester Provincial Hospital in Worcester, a town of approximately 230 000 in a farming community 120 kilometers outside Cape Town. WMOU

For peer review only - http://bmjopen.bmj.com/site/about/guidelines.xhtml

provides delivery services for ~3600 women annually. The antenatal HIV prevalence is approximately 16%. Women requiring more advanced care are referred to Worcester (secondary) and Tygerberg (tertiary) Hospitals. The community is structurally disadvantaged, and many depend on seasonal employment on farms. In both areas the population is mobile; women move within the WC province and may deliver outside the proscribed referral axes.

Enrolment started at GMOU in Cape Town in September 2016 and at WMOU in January 2018.

All women seeking care at the sentinel primary-care sites were included. Most women who use public maternity services, including those with medical and obstetric complications, initially present to primary care, therefore **situating enrolment at the primary-care facility allowed us to capture a sample representative of the pregnant population in the geographic drainage area of that facility.**

Maternal and Child Health Services in the Western Cape

Obstetric care is free at the point of service and approximately 65% of women present at/before 20 weeks gestation[24]. Antenatal care for uncomplicated pregnancies is provided at Basic Antenatal Clinics and MOUs, the latter able to manage uncomplicated vaginal deliveries. At any stage during pregnancy or peri-partum women can be referred to district, regional or tertiary hospitals according to standard operating procedures. HIV testing is routine at timepoints throughout gestation and WLHIV are initiated/re-initiated on ART[25]; those already receiving ART may transfer their HIV care to the MOU. Clients with other underlying medical conditions (e.g., pre-existing hypertension, diabetes mellitus, cardiac conditions) and/or who develop pregnancy-related medical conditions (e.g., hypertensive disorders of pregnancy, gestational diabetes) continue antenatal care at hospital. The MOU dispenses ART and antenatal supplements and preventive therapies recommended by the WHO

For peer review only - http://bmjopen.bmj.com/site/about/guidelines.xhtml

in pregnancy (i.e. iron and folate supplements, tetanus and influenza vaccines)[26]. Midwives treat the common complaints of pregnancy (heartburn, nausea), urinary tract infection, vaginal candidiasis and provide syndromic treatment for sexually transmitted infections (STI). Frequently, these medicines are dispensed directly from *ward-stock* without a linked digital record, although details are recorded in paper-based registers.

Within resource constraints, the WC endeavors to provide an antenatal ultrasound scan to clients before 22 weeks gestation for determining gestational age. If concerns are identified women are up-referred for formal fetal anomaly review.

Antenatal visits, HIV testing, transfers and deliveries are recorded against patient names in individual paper-based registers. Monthly aggregate statistics of key obstetric indicators (Table 1) are manually counted from these registers and submitted centrally as part of the routine District Health Information System platform.

Follow-up

The Maternity Case Record (MCR) is a patient-held paper-based document distributed at the first antenatal visit that serves as a record of all clinical obstetric care until discharge after pregnancy outcome, regardless of level of care. It is utilized throughout South Africa and archived at the site of outcome. Chronic medication and any agents dispensed during pregnancy should be recorded in the MCR by the attending clinicians. However, medicines received at specialist clinics, during hospital admissions and over-the-counter medicines are often not documented [3, 22].

For peer review only - http://bmjopen.bmj.com/site/about/guidelines.xhtml

After birth, live- and still-born neonates are examined by the attending clinician (nurse midwife/doctor) and the outcome of the limited neonatal surface examination is recorded in the MCR. This examination has been shown to detect most major congenital malformations in neonates, i.e. those that are visible and do not require diagnostic tools[27]. At GMOU, a clinician employed by the PER performs a review of clinical records to obtain additional data for congenital disorders and stillbirths. In the case of stillbirth, the placenta may be sent for histological examination.

In the WC, most women (99%) give birth at a health facility[24]. Those who do not, will bring their infants to the MOU soon after birth for review and registration.

For the purposes of the PER, the MCR serves as the primary source of prospectively-collected clinical data. Thus, women enter the cohort on first visit to the MOU and are followed up until pregnancy outcome.

Data collection

The PER digitizes routinely-collected data from the clinical stationery if not already digitized under existing service delivery. In addition to the patient-held MCR, data sources include primary-care dating ultrasound reports, and the STI and labour ward delivery registers. As we are using operational data, definitions have been aligned with operational clinical definitions in the WC. Using other routinely collected data elements (gestational age, neonate anthropometry) we are able to align case definitions with those of the Global Alignment of Immunization Safety Assessment in Pregnancy[28], allowing for harmonization of data and meaningful comparisons with equivalent datasets. Additionally, we collect or calculate health indicators for the routine monthly aggregate reports required by the MOUs (Table 1).

For peer review only - http://bmjopen.bmj.com/site/about/guidelines.xhtml

Externally-funded PER data clerks are embedded at the facilities and project-augmented data collection is accommodated within the routine patient and document flow without disruption of clinical care.

The provincial government of the WC operates as a single provider of public sector health services. A 9-digit numeric folder number which is common across the health platform for a given patient facilitates the harmonization of all electronic health records within the Provincial Health Data Centre (PHDC), the information exchange that consolidates all electronic administrative, pharmacy, laboratory, and disease-specific information[21]. PER data are recorded against this identifier and contribute to the PHDC.

All MOUs use the Primary Health Care Information Service (PHCIS) electronic medical records system which records attendance against patient identifiers, and ART in WLHIV. PHCIS automatically generates a unique folder number for live infants at birth, providing electronic linkage between mother and baby. Clinicom performs this function at all hospitals. Data are imported daily by the PHDC[21].

Completeness of Medicine Exposure data

Electronic dispensing data in the PHDC is augmented by the PER which captures medicine exposures elicited from the women during the clinical consultation and ward-stock medicines recorded by clinicians in the MCR. The PER also records some lifestyle factors (weight gain, alcohol, tobacco, recreational drugs) that may act as confounders for certain outcomes. Combining the electronic pharmacy data in the PHDC strengthens the ascertainment of exposures, providing a complete list of medication dispensed from public sector pharmacies. Using multiple data sources for this has been

For peer review only - http://bmjopen.bmj.com/site/about/guidelines.xhtml

shown to provide a more complete picture of antenatal medicine use essential for pregnancy exposure research[3, 29, 30].

Outcome Ascertainment

Information on neonatal outcomes such as vital status, birth weight, gestational age, and APGAR scores tend to be consistently captured across the cohort. The key findings of the neonatal surface examinations, although often perfunctory, usually result in the recording of notable physical anomalies. Internal anomalies such as cleft palate, hip dysplasias, and cardiac anomalies as well as more subtle dysmorphic features may be missed at the time of the initial neonatal examination. Details of neonatal deaths, and major congenital disorders often require review of inpatient records at the delivery facilities.

PER data are imported daily into the PHDC and linked using patient identifiers, providing a comprehensive electronic clinical record at the level of the individual which is accessible to the attending clinicians. Both systems benefit greatly from this design. The PER allows for validation of the provincial dataset as relates to pregnancy and delivery, and the PHDC is able to identify missing outcomes (often at sites outside the referral axes) or exposures (from electronic pharmacy dispensing) not included in the PER.

Findings to date

Between 01 September 2016 and 31 August 2021, 31 346 pregnancies were recorded in the PER. To assess robustness of the dataset, we analysed data for a subset of women who attended their first visit to antenatal care between 01 January 2018 and 31 December 2019 (Table 2). Over this two-year period, 14 527 individual pregnancies were recorded in the PER: 9435 and 5092 at the urban and rural

For peer review only - http://bmjopen.bmj.com/site/about/guidelines.xhtml

site, respectively. Outcomes were determined for 93.4% of pregnancies (n = 13 574). Gestational dating scans were performed in 38.5% (n = 5583) of all enrolees, of whom 60% (n = 3345) were ≤22 weeks, facilitating more precise gestational dating at birth as well as timing of exposures. Overall, 1287 women (9%) were exposed to a potentially unsafe medicine over the course of their pregnancies (Table 3 and Supplementary Table 1).

Where relevant, we compared rates of key adverse birth outcomes in the PER with official aggregate routine indicator data for the WC[24, 31-33], derived from register aggregates reported through the District Health Information System (Table 3). At the urban site, 38 congenital disorders were confirmed in 2018 – 2019 (Table 4). Twelve were classified as minor (pre-axial polydactyly, undescended testes, subglottic stenosis not requiring intervention). Major congenital disorders included two cases of fetal hydantoin syndrome (both diagnosed antenatally); and four neural tube defects (two identified antenatally and two at birth). The congenital disorder data are still being cleaned for analysis with pregnancy outcomes.

Published and other outputs

We conducted an initial baseline assessment comparing clinical records to dispensing data before the implementation of the PER[22] and recently updated the analysis demonstrating the value of combining PER and electronic pharmacy data in improving medicine exposure ascertainment[3]. We are currently investigating the impact of data source on gestational age (Malaba T, manuscript in preparation) and hypertensive disorders of pregnancy[34]. PER data have contributed to population-based analyses describing the use and safety of sodium valproate and isoniazid for TB preventive therapy in pregnancy[35, 36]. In addition, initiation of the PER provided the opportunity to host a workshop, *Building Teratovigilance Capacity in Africa,* which provided networking and training

For peer review only - http://bmjopen.bmj.com/site/about/guidelines.xhtml

opportunities to 60 delegates from sub-Saharan Africa https://globalpharmacovigilance.tghn.org/resources/building-teratovigilance-capacity-africa/.

System strengthening

In addition to the employment of project-specific staff, embedded with computers at the facilities, the project supports on-going training of clinical staff to improve and standardize clinical history-taking with an emphasis on exposures, neonatal examination and clinical record keeping. Open resources include the WHO/TDR *Stepwise Surface Examination of the Newborn* (https://www.who.int/tdr/publications/videos/stepwise-surface-examination-newborns/en/) and the training modules for midwives we developed as part of the South African National Pregnancy Exposure Registry (https://www.ubomibuhle.org.za/training-lessons)[37]. These resources are freely available and are now in use at PER sites across South Africa.

Strengths and Weaknesses

Strengths

The integration of the PER within the PHDC greatly increases the completeness of the data. It facilitates identification of pregnancy outcomes at facilities outside our sentinel referral chains reducing loss to follow-up. Harmonization and triangulation of two data sources for medicine exposures (i.e., clinical records and electronic pharmacy records) provides a more robust summary of exposures than either alone[3, 17, 22]. These systems comprise unique infrastructure able to address clinical and public health concerns in a low-middle income setting.

For peer review only - http://bmjopen.bmj.com/site/about/guidelines.xhtml

Accurate timing of exposures over the course of pregnancy is crucial to assess potential associations with adverse pregnancy outcomes. Collecting multiple reference points for gestational age (i.e., neonatal record, ultrasound, last menstrual period, symphysis-fundal height) enabled the development of a hierarchy of methods and the allocation of a confidence score to the reported gestational age[38-40]. This offers an advantage over insurance claims datasets which are often used to determine safety information and in which pregnancy and gestational age must be inferred from clinical coding alone.

In line with WHO recommendations[20], all women attending the PER primary care sites are enrolled and we reflect background rates of important pregnancy parameters similar to what is expected from national aggregate data. This will be expanded to include background rates for congenital disorders, data which are lacking in South Africa[41]. This structure also allows for the analysis of multiple current and potential future exposures and emerging health concerns e.g., novel medicines and vaccines such as for SARS-CoV-2.

From the outset, it was important to avoid a parallel system and support project sustainability. The PER has been integrated into the existing clinical and clerical routines and uses local electronic health information platforms. It allows for electronic generation of key monthly indicators at primary care sites that are otherwise collected by hand.

As the cohort expands, capacity to conduct nested studies that facilitate signal detection and signal verification of potential or suspected teratogens will improve. The collection of individual-level data in a large prospectively enrolled cohort, representative of both urban and rural WC populations who

For peer review only - http://bmjopen.bmj.com/site/about/guidelines.xhtml

For peer review only

use public sector services will support more robust analyses that can better account for confounding factors in such observational data.

Weaknesses

The PER digitizes clinical data recorded in operational stationery and we cannot control for data quality nor account for missing data in the source documents. To address this, we have engaged in on-going training at the sites with an emphasis on drug history taking, medical record-keeping and neonatal examination offering in-person teaching and video tutorials. Clinical staff have been provided with Medicine Identification Aids with photographs of common formulations and packaging, and the WHO Birth Defects atlas[42]. However, misclassification remains a potential risk.

Notwithstanding the advantages of the individual-level data available within the PHDC, data are limited to those that are entered into one of the electronic medical records systems used in the public sector. In terms of medicine exposures, the PER documents dispensed medication which may not reflect actual use. In addition, medicines obtained outside of the public sector systems, from private doctors or over-the-counter from pharmacies are not included unless they are noted in the clinical records[3]. Similarly, traditional and complementary medicines lack a linked electronic footprint and are not included.

The PER database is parsimonious by design and necessity and we are unable to account for unmeasured confounders. However, data fields are collected for the entire cohort who are all drawn from the same geographical areas served by the primary care clinics. Additionally, we record limited data on lifestyle factors relevant in pregnancy (weight gain, exposure to tobacco, alcohol, recreational drugs) which are lacking from equivalent population datasets based on insurance claims data.

For peer review only - http://bmjopen.bmj.com/site/about/guidelines.xhtml

Collaboration

As with the PHDC within which it is located, the PER can address clinical, operational and research needs, and data access is specific to each. Aggregate reports are available to managers. Data are anonymised using standard protocols for de-identifying records before they are shared with researchers who are not directly engaged in the women's clinical care. It is anticipated that such de-identified individual-level data may be shared as part of the South African National Pregnancy Registry[37] and with similar PER initiatives regionally or internationally[43]. Data-sharing commitments are particularly relevant to research of rare events such as congenital disorders[20]. The PHDC has in-built privacy systems and strict governance structures managing the protection and use of health data for both service and research purposes and these apply to the PER[21].

Patient and public involvement

The PER is integrated into the data collection and curation services of the Western Cape Government Department of Health and clinical and other service providers have engaged with the project since its inception. The data are available to managers as aggregate reports and to contribute to the electronic clinical records accessible by clinicians. Feedback from users contributes iteratively to optimization of the PER to improve health outcomes for pregnant women and infants.

Conclusions

Research on medicine safety in pregnancy requires data on individual pregnancies, mother-infant linkage, medication exposure, gestational age at exposure, and maternal and birth outcomes. Data completeness and robustness continues to improve with on-going training, evolution of routine clinical information systems, and increasing political focus on pregnancy exposures. The cohort is well-placed to detect large signals in pregnancy outcomes as novel maternal exposures are introduced, and

For peer review only - http://bmjopen.bmj.com/site/about/guidelines.xhtml

to contribute to cohort harmonization for rarer outcomes and address the lack of information on congenital disorders in Africa.

Ethics approval

The WC PER has been approved by the Faculty of Health Sciences Human Research Ethics Committees of the University of Cape Town (HREC: 749/2015) and Stellenbosch University (N17/04/040, N20/08/084), and the Western Cape Government Health Research Committee. The requirement for individual informed consent was waived by the ethics committee and all data were anonymized before being transferred for analysis.

Funding

This work was supported by the US National Institutes of Health [R01HD080465, U01AI069911, U01AI069924, K43TW010683], Bill and Melinda Gates Foundation [INV-004508; 1164272; 1191327; INV-004657], the Wellcome Trust [203135/Z/16/Z], the International AIDS Society [2017-518/SLO], the United States Agency for International Development [72067418CA00023] and the US Centers for Disease Control and Prevention [GH001934].

Acknowledgements

We acknowledge and thank the PER data teams in Cape Town and Worcester, and the clinical and management staff at the facilities. The support and investment of the subdistrict and provincial health management is crucial to maintaining the initiative. We further acknowledge colleagues from the Western Cape Government: Health and the City of Cape Town.

For peer review only - http://bmjopen.bmj.com/site/about/guidelines.xhtml

Author contributions

Conception and design: EK, UM, AB, ALS, MAD, LM

Design and Implementation including data systems: EK, UM, ALS, GP, KF, JE

Data harmonization: AH, FP, JE, AB, MAD, EK

Clinical oversight: GP, MK, CS, NR, SG, AO, KF

Data cleaning & analysis: EK, ALS, UM, KA, AH

All authors critically reviewed the manuscript.

Conflict of interests

EK, AB, MAD and KA received funding from Viiv Healthcare unrelated to this project.

The authors declare no conflicts of interest.

For peer review only

For peer review only - http://bmjopen.bmj.com/site/about/guidelines.xhtml

References

1. Daw JR, Hanley GE, Greyson DL, Morgan SG. Prescription drug use during pregnancy in developed countries: a systematic review. Pharmacoepidemiol Drug Saf. 2011;20(9):895-902.
2. Mohseni M, Azami-Aghdash S, Gareh Sheyklo S, Moosavi A, Nakhaee M, Pournaghi-Azar F, et al. Prevalence and Reasons of Self-Medication in Pregnant Women: A Systematic Review and Meta-Analysis. Int J Community Based Nurs Midwifery. 2018;6(4):272-84.
3. van der Hoven J, Allen E, de Waal R, Cois A, Maartens G, Myer L, et al. Antenatal medicine use in South African women: a comparison of three methods of ascertainment. BMC Pregnancy Childbirth. 2022;(under review).
4. Zash R, Holmes L, Diseko M, Jacobson DL, Brummel S, Mayondi G, et al. Neural-Tube Defects and Antiretroviral Treatment Regimens in Botswana. N Engl J Med. 2019;381(9):827-40.
5. Zash R, Makhema J, Shapiro RL. Neural-Tube Defects with Dolutegravir Treatment from the Time of Conception. N Engl J Med. 2018;379(10):979-81.
6. Gupta A, Montepiedra G, Aaron L, Theron G, McCarthy K, Bradford S, et al. Isoniazid Preventive Therapy in HIV-Infected Pregnant and Postpartum Women. N Engl J Med. 2019;381(14):1333-46.
7. Bianchi DW, Kaeser L, Cernich AN. Involving Pregnant Individuals in Clinical Research on COVID-19 Vaccines. JAMA. 2021;325(11):1041-2.
8. World Health Organization. Rapid Communication on forthcoming changes to the programmatic management of tuberculosis preventive treatment Geneva: World Health Organization; 2020 [Available from: https://www.who.int/publications/m/item/WHO-UCN-TB-2020.4.] (Accessed 17 March 2022)
9. World Health Organization. CONSOLIDATED GUIDELINES ON HIV PREVENTION, TESTING, TREATMENT, SERVICE DELIVERY AND MONITORING: RECOMMENDATIONS FOR A PUBLIC HEALTH APPROACH Geneva: World Health organization; 2021 [Available from: https://www.who.int/publications/i/item/9789240031593.] (Accessed 17 March 2022)
10. World Health Organization. COVID-19 vaccines: safety surveillance manual, second edition Geneva: World Health Organization; 2021 [Available from: https://www.who.int/publications/i/item/10665345178.] (Accessed 17 March 2022)
11. Craig AM, Hughes BL, Swamy GK. Coronavirus disease 2019 vaccines in pregnancy. Am J Obstet Gynecol MFM. 2021;3(2):100295.
12. Gupta A, Hughes MD, Garcia-Prats AJ, McIntire K, Hesseling AC. Inclusion of key populations in clinical trials of new antituberculosis treatments: Current barriers and recommendations for pregnant and lactating women, children, and HIV-infected persons. PLoS Med. 2019;16(8):e1002882.
13. Moodley J, Ngene NC, Khaliq OP, Hunter M. An imperative to offer pregnant and lactating women access to the COVID-19 vaccination roll-out programme. S Afr Med J. 2021;111(6):567-9.
14. World Health Organization, International Maternal Pediatric Adolescent AIDS Clinical Trials Network. APPROACHES TO ENHANCE AND ACCELERATE STUDY OF NEW DRUGS FOR HIV AND ASSOCIATED INFECTIONS IN PREGNANT WOMEN. Geneva: World Health Organization; 2021. [https://www.who.int/publications/i/item/9789240040182]. (Accessed 17 March 2022)
15. Holmes LB, Wyszynski DF. North American antiepileptic drug pregnancy registry. Epilepsia. 2004;45(11):1465.
16. Scheuerle A, Covington D. Clinical review procedures for the Antiretroviral Pregnancy Registry. Pharmacoepidemiol Drug Saf. 2004;13(8):529-36.
17. Gelperin K, Hammad H, Leishear K, Bird ST, Taylor L, Hampp C, et al. A systematic review of pregnancy exposure registries: examination of protocol-specified pregnancy outcomes, target sample size, and comparator selection. Pharmacoepidemiol Drug Saf. 2017;26(2):208-14.
18. Berrueta M, Ciapponi A, Bardach A, Cairoli FR, Castellano FJ, Xiong X, et al. Maternal and neonatal data collection systems in low- and middle-income countries for maternal vaccines active safety surveillance systems: A scoping review. BMC Pregnancy Childbirth. 2021;21(1):217.

For peer review only - http://bmjopen.bmj.com/site/about/guidelines.xhtml

19. Mehta U, Kalk E, Fairlie L, Boulle A, Rees H. Why South Africa urgently needs to support the development of pregnancy exposure registries. S Afr Med J. 2019;109(5):294-5.
20. Mehta U, Clerk C, Allen E, Yore M, Sevene E, Singlovic J, et al. Protocol for a drugs exposure pregnancy registry for implementation in resource-limited settings. BMC Pregnancy Childbirth. 2012;12:89.
21. Boulle A, Heekes A, Tiffin N, Smith M, Mutemaringa T, Zinyakatira N, et al. Data Centre Profile: The Provincial Health Data Centre of the Western Cape Province, South Africa. Int J Popul Data Sci. 2019;4(2):1143.
22. Mehta U, Heekes A, Kalk E, Boulle A. Assessing the value of Western Cape Provincial Government health administrative data and electronic pharmacy records in ascertaining medicine use during pregnancy. S Afr Med J. 2018;108(5):439-43.
23. Okoli GN, Myles P, Murray-Thomas T, Shepherd H, Wong ICK, Edwards D. Use of Primary Care Data in Research and Pharmacovigilance: Eight Scenarios Where Prescription Data are Absent. Drug Saf. 2021.
24. Day C, Gray C, Padayachee T, Cois A. Chapter 20, Health and Related Indicators. South African Health Review 2020. 2020.
25. South African Department of Health. Guideline for the Prevention of Mother to Child Transmission of Communicable Infections. In: Health, editor. PretoriaNovember 2019.
26. World Health Organization. WHO recommendations on antenatal care for a positive pregnancy experience. Geneva: WHO Library Cataloguing-in-Publication Data; 2016.
27. Holmes LB, Nasri HZ, Hunt AT, Zash R, Shapiro RL. Limited surface examination to evaluate potential teratogens in a resource-limited setting. Birth Defects Res. 2021;113(9):702-7.
28. Bonhoeffer J, Kochhar S, Hirschfeld S, Heath PT, Jones CE, Bauwens J, et al. Global alignment of immunization safety assessment in pregnancy - The GAIA project. Vaccine. 2016;34(49):5993-7.
29. Andrade SE, Berard A, Nordeng HME, Wood ME, van Gelder M, Toh S. Administrative Claims Data Versus Augmented Pregnancy Data for the Study of Pharmaceutical Treatments in Pregnancy. Curr Epidemiol Rep. 2017;4(2):106-16.
30. Grzeskowiak LE, Gilbert AL, Morrison JL. Investigating outcomes associated with medication use during pregnancy: a review of methodological challenges and observational study designs. Reprod Toxicol. 2012;33(3):280-9.
31. Massyn N, Day C, Ndlovu N, Padayachee T. District Health Barometer 2019/2020. Durban: Health Systems Trust; 2020.
32. National Perinatal Mortality and Morbidity Committee. NAPEMMCO Trennial Report 2017 - 2019. 2017-2019.
33. Provincial Perinatal Morbidity and Mortality Commitee. Western Cape Saving Babies Trennial Report 2017 -2019. 2020.
34. Slogrove A, Davies MA, Williams P, Abrams E, Theron G, Cotton M, et al., editors. Hypertensive Disorders in Pregnancy and HIV: A Province-wide Cohort Analysis during 2018 and 2019 in the Western Cape, South Africa. 13th International Workshop on HIV Pediatrics; 2021 16-17 July 2021; virtual.
35. Kalk E, Heekes A, Mehta U, de Waal R, Jacob N, Cohen K, et al. Safety and Effectiveness of Isoniazid Preventive Therapy in Pregnant Women Living with Human Immunodeficiency Virus on Antiretroviral Therapy: An Observational Study Using Linked Population Data. Clin Infect Dis. 2020;71(8):e351-e8.
36. Mehta U, Smith M, Kalk E, Hayes H, Swart A, Tucker L, et al. Understanding and Responding to Prescribing Patterns of Sodium Valproate-Containing Medicines in Pregnant Women and Women of Childbearing Age in Western Cape, South Africa. Drug Saf. 2021;44(1):41-51.
37. Buhle. U. Understanding Birth Outcomes for Mothers and Infants: Building Healthcare by Linking Exposures 2021 [Available from: https://www.ubomibuhle.org.za/.]
38. Geerts L, Poggenpoel E, Theron G. A comparison of pregnancy dating methods commonly used in South Africa: a prospective study. S Afr Med J. 2013;103(8):552-6.

For peer review only - http://bmjopen.bmj.com/site/about/guidelines.xhtml

39. Malaba TR, Newell ML, Madlala H, Perez A, Gray C, Myer L. Methods of gestational age assessment influence the observed association between antiretroviral therapy exposure, preterm delivery, and small-for-gestational age infants: a prospective study in Cape Town, South Africa. Ann Epidemiol. 2018;28(12):893-900.
40. Quinn JA, Munoz FM, Gonik B, Frau L, Cutland C, Mallett-Moore T, et al. Preterm birth: Case definition & guidelines for data collection, analysis, and presentation of immunisation safety data. Vaccine. 2016;34(49):6047-56.
41. Lebese V, Aldous C, Malherbe HL. South African congenital disorders data, 2006 - 2014. S Afr Med J. 2016;106(10):992-5.
42. WHO/CDC/ICBDSR. Birth defects surveillance: atlas of selected congenital anomalies. Geneva: World Health Organization; 2014.
43. AIeDEA-East Africa. Measuring Adverse Pregnancy & Newborn Outcomes (MANGO): an IedEA Collaboration Study 2021 [Available from: https://globalhealth.iu.edu/research/iedea-main-page/.]

For peer review only

For peer review only - http://bmjopen.bmj.com/site/about/guidelines.xhtml

Table 1. Summary of Data Elements in the PER

Variables collected	Calculated parameters	MOU aggregate statistics
Antenatal<br>Maternal date of birth<br>Date of first antenatal visit<br>Last menstrual period<br>Parity, Gravidity<br>Obstetric & Medical history<br>Chronic medication<br>Height, mid-upper arm circumference, weight, blood pressure, urinalysis<br>Symphysis fundal height<br>Alcohol, tobacco, drug use<br>Number of antenatal visits	Gestational age at first antenatal visit	Number of first visits<br>Number of women first ANC < 20 weeks<br>Number age < 20 years or > 38 years<br>Number grand multipara (≥ 5 deliveries)<br>Number high blood pressure/proteinuria
Vertical transmission of HIV<br>HIV status at first antenatal visit<br>Subsequent positive HIV test<br>HIV treatment incl. regimen switches<br>CD4 count<br>Viral load<br>HIV-exposed infant HIV-PCR	Number of women at high risk of vertical HIV transmission [18]<br>ART in hand at estimated time of conception<br>ART in hand at delivery	Number of women living with HIV:<br>Before pregnancy<br>During pregnancy<br>Number of women on ART (1st & 2nd line):<br>Before, during pregnancy<br>VL unsuppressed at pregnancy & delivery<br>Number of infant birth HIV-PCR
Ultrasound<br>Gestational age<br>Abnormalities<br>Expected date of delivery		Number of ultrasounds conducted<br>Number multiple pregnancies
Maternal outcome<br>Facility-based death	Vital status	Maternal death
Peri-partum<br>Date & site of outcome		Number of deliveries

For peer review only

Method of delivery<br>Gestational age at outcome	Prematurity (< 37 completed weeks gestation)	
Pregnancy outcome<br>Livebirth<br>Stillbirth<br>Miscarriage<br>Termination of pregnancy<br>Ectopic pregnancy<br>Molar pregnancy	Gestational age at pregnancy outcome	Number of livebirths, stillbirths, miscarriages
Neonate<br>Date of birth<br>Sex, APGAR scores<br>Gestational age<br>Birth weight, length, head circumference, foot length<br>Neonatal surface examination<br>Abnormalities noted	Gestational age at birth<br>Low birth weight (< 2500g)<br>Prematurity (< 37 completed weeks gestation)<br>Neonatal death	Number of low birth weight infants<br>Number premature infants<br>Number neonatal deaths<br>Perinatal mortality rate

ANC – antenatal care; ART – antiretroviral therapy; HIV – Human Immune Deficiency Virus; MOU – midwife obstetric unit; PCR – polymerase chain reaction

For peer review only

For peer review only - http://bmjopen.bmj.com/site/about/guidelines.xhtml

Table 2. Maternal and obstetric characteristics of the cohort 2018-2019

Variable	PER total n = 14 527	PER urban n = 9435 (65%)	PER rural n = 5092 (35%)
Age (years) median (IQR)	27 (23 – 32)	28 (23 – 33)	26 (22 – 31)
Living with HIV at pregnancy outcome	3931 (27.1)	3241 (34.3)	690 (13.6)
Obstetric ultrasound present n (%)<br>Early ultrasound (i.e. < 22 weeks) n (% of US)	5583 (38.4)<br>3345 (59.9)	4063 (43.1)<br>2393 (58.9)	1520 (29.9)<br>952 (62.6)
Potentially unsafe medicine exposure	1287 (9.0)	857 (9.3)	430 (8.5)
Gestational age at birth (weeks) median (IQR)	40 (37 – 40)	40 (36 – 40)	39 (35 – 40)
Birth weight (grams) median (IQR)	3100 (2750 – 3440)	3140 (2800 – 3480)	2975 (2575 – 3320)
Low birth weight[a] n (%)	1736 (12.0)	879 (9.3)	857 (16.8)
Premature birth[b] n(%)	2949 (20.3)	1735 (18.4)	1214 (23.8)
Pregnancy outcome n (%)			
Live birth	12 419 (85.5)	1189 (82.3)	4630 (90.9)
Still birth	296 (2.0)	180 (1.9)	116 (2.3)
Neonatal death[c]	109 (0.8)	71 (0.5)	36 (0.7)
Miscarriage	395 (2.7)	318 (3.4)	77 (1.5)
Ectopic pregnancy	82 (0.6)	60 (0.6)	22 (0.4)
Termination of pregnancy	273 (1.9)	223 (2.4)	50 (1.0)
Unknown	953 (6.6)	792 (8.4)	161 (3.1)
Delivery method[d] n(%)			
Born before arrival at birthing facility	608 (4.7)	245 (3.1)	363 (7.6)
Vaginal delivery	7587 (59.2)	4655 (57.9)	2932 (61.3)
Assisted delivery[e]	140 (1.1)	51 (0.6)	89 (1.9)
Caesarean section	3416 (26.6)	2411 (30.0)	1005 (21.0)
Unknown	1073 (8.4)	680 (8.5)	393 (8.2)
Infant outcome[d] n(%)			
Stillborn	296 (2.3)	180 (2.2)	116 (2.4)
Early neonatal death[c]	80 (0.6)	55 (0.7)	25 (0.5)
Late neonatal death	29 (0.2)	18 (0.2)	11 (0.2)
Alive	12 419 (96.8)	7798 (96.9)	4630 (96.8)
Tobacco use[f] n(%)			
Current user	1297 (8.9)	87 (0.9)	1210 (23.8)
Past user	55 (0.4)	13 (0.1)	42 (0.8)
Never user	9997 (68.8)	7222 (76.5)	2775 (54.5)
Not reported	3178 (21.9)	2113 (14.5)	1065 (7.3)
Alcohol use[f] n(%)			
Current user	588 (4.1)	339 (3.6)	249 (4.9)
Past user	167 (1.2)	66 (0.7)	101 (2.0)
Never user	10 570 (72/8)	6885 (73.0)	3685 (72.4)
Not reported	3202 (22.0)	2145 (14.8)	1057 (7.3)

[a] birthweight<2500g; liveborn infants only

[b] birth < 37 completed weeks gestation; liveborn infants only

For peer review only - http://bmjopen.bmj.com/site/about/guidelines.xhtml

[c] neonatal death: death before 28 days of life; early neonatal death: death before 7days of life; late neonatal death: death between 8 and 28 days of life

[d] viable pregnancies (i.e >27 weeks gestation(17)) (n=12 824)

[e] forceps or vacuum delivery

[f] reported at first antenatal visit

For peer review only

Table 3. Comparison between PER reported or calculated PER outcomes and aggregate indicators in formal provincial information systems

Indicator	PER total n = 14 527	PER urban n = 9435 (65%)	PER rural n = 5092 (35%)	Reported aggregate for the Western Cape 2017 - 2019[a]
Still birth[b] n (%)<br><br>Per 1000 births	296 (2.0)<br><br>20.0	180 (1.9)<br><br>19.1	116 (2.4)<br><br>24.0	2.2%[26]<br><br>18.5[17]<br>22.1[25,26]
Neonatal death in facility rate[c] per 1000 live births	8.7	9.2	7.7	8.9[17,25]
Perinatal mortality rate[d] per 1000 births	29	29	29	25.6[17]<br>27.9[25]<br>29.1[26]
Low birth weight[e] n(%)	1737 (12.0)	879 (9.3)	857 (16.8)	14.9% urban subdistrict<br>18.4% rural subdistrict[26]
Maternal mortality in facility ratio per 100 000 live births		63.5	Insufficient data	43.6 – 66.8[25]
Teenage pregnancies (10 – 19 years) n(%)	929 (6.4)	450 (4.8)	497 (9.4)	3.5% urban subdistrict<br>7.3% rural subdistrict[26]
Caesarean section rate per 1000 births	3416 (26.6)	2411 (30.0)	1005 (21.0)	28.9[25] - 29.3[26]

[a] includes aggregate reports compiled from the District Health Information System and Perinatal Problem Identification Programme[17, 25-26]

[b] delivery of a baby with no signs of life after 27 completed weeks of gestation (i.e., viable baby born dead)

[c] death before 28 days of life

[d] still birth plus neonatal deaths <8 days per 1000 births

[e] birthweight<2500g; liveborn infants only

For peer review only - http://bmjopen.bmj.com/site/about/guidelines.xhtml

Table 4. Congenital Disorders in PER 2018 – 2019; urban site only (alphabetical)

	Disorder	Number
Major disorders	Cardiac	1
	Cleft palate	2
	Congenital diaphragmatic hernia	2
	CNS excluding neural tube defect	2
	Fetal alcohol syndrome	1
	Fetal hydantoin syndrome	2
	Hypospadias	2
	Jejunal atresia	1
	Neural tube defect	4
	Not otherwise specified	1
	Omphalocoele	1
	Renal	1
	Recto-membranous urethral fistula	1
	Skeletal dysplasia	1
	Trisomies<br>T21<br>T13	 2<br>1
Minor disorders	Pre-axial polydactyly	8
	Subglottic stenosis (not requiring intervention)	1
	Undescended testes	3

CNS – central nervous system

For peer review only

Supplementary Table 1. Potentially unsafe medicines, excluding ART, identified in the PER over the course of gestation (alphabetical)

**Name**
Carbamazepine
Carbimazole
Diazepam
Doxycycline
Enalapril
Gentamicin
Ibuprofen
Lithium
Losartan
Phenytoin
Sulfamethoxazole & trimethoprim
Valproate
Warfarin

ART – antiretroviral therapy

For peer review only

STROBE Statement—Checklist of items that should be included in reports of ***cohort studies***

	Item No	Recommendation	page
**Title and abstract**	1	(*a*) Indicate the study's design with a commonly used term in the title or the abstract	1
		(*b*) Provide in the abstract an informative and balanced summary of what was done and what was found	3
**Introduction**			
Background/rationale	2	Explain the scientific background and rationale for the investigation being reported	5-7
Objectives	3	State specific objectives, including any prespecified hypotheses	7
**Methods**			
Study design	4	Present key elements of study design early in the paper	7-8
Setting	5	Describe the setting, locations, and relevant dates, including periods of recruitment, exposure, follow-up, and data collection	7 - 10
Participants	6	(*a*) Give the eligibility criteria, and the sources and methods of selection of participants. Describe methods of follow-up	8-9
		(*b*) For matched studies, give matching criteria and number of exposed and unexposed	N/A
Variables	7	Clearly define all outcomes, exposures, predictors, potential confounders, and effect modifiers. Give diagnostic criteria, if applicable	11-12, 16
Data sources/ measurement	8*	For each variable of interest, give sources of data and details of methods of assessment (measurement). Describe comparability of assessment methods if there is more than one group	11-12 Table 1
Bias	9	Describe any efforts to address potential sources of bias	14-16
Study size	10	Explain how the study size was arrived at	N/A
Quantitative variables	11	Explain how quantitative variables were handled in the analyses. If applicable, describe which groupings were chosen and why	12-13 Tables 2-4
Statistical methods	12	(*a*) Describe all statistical methods, including those used to control for confounding	Cohort *description;* limited analyses
		(*b*) Describe any methods used to examine subgroups and interactions	N/A
		(*c*) Explain how missing data were addressed	14-16
		(*d*) If applicable, explain how loss to follow-up was addressed	16
		(*e*) Describe any sensitivity analyses	12-13
**Results**			
Participants	13*	(a) Report numbers of individuals at each stage of study—eg numbers potentially eligible, examined for eligibility, confirmed eligible, included in the study, completing follow-up, and analysed	12-13
		(b) Give reasons for non-participation at each stage	N/A
		(c) Consider use of a flow diagram	
Descriptive data	14*	(a) Give characteristics of study participants (eg demographic, clinical, social) and information on exposures and potential confounders	12-13 Tables 2-4
		(b) Indicate number of participants with missing data for each variable of interest	N/A
		(c) Summarise follow-up time (eg, average and total amount)	N/A
Outcome data	15*	Report numbers of outcome events or summary measures over time	12-13

For peer review only - http://bmjopen.bmj.com/site/about/guidelines.xhtml

			Tables 2-4
Main results	16	(*a*) Give unadjusted estimates and, if applicable, confounder-adjusted estimates and their precision (eg, 95% confidence interval). Make clear which confounders were adjusted for and why they were included	N/A
		(*b*) Report category boundaries when continuous variables were categorized	N/A
		(*c*) If relevant, consider translating estimates of relative risk into absolute risk for a meaningful time period	
Other analyses	17	Report other analyses done—eg analyses of subgroups and interactions, and sensitivity analyses	N/A
**Discussion**			
Key results	18	Summarise key results with reference to study objectives	13-14
Limitations	19	Discuss limitations of the study, taking into account sources of potential bias or imprecision. Discuss both direction and magnitude of any potential bias	16
Interpretation	20	Give a cautious overall interpretation of results considering objectives, limitations, multiplicity of analyses, results from similar studies, and other relevant evidence	17-18
Generalisability	21	Discuss the generalisability (external validity) of the study results	14, 17- 18
**Other information**			
Funding	22	Give the source of funding and the role of the funders for the present study and, if applicable, for the original study on which the present article is based	18

*Give information separately for exposed and unexposed groups.

Note: An Explanation and Elaboration article discusses each checklist item and gives methodological background and published examples of transparent reporting. The STROBE checklist is best used in conjunction with this article (freely available on the Web sites of PLoS Medicine at http://www.plosmedicine.org/, Annals of Internal Medicine at http://www.annals.org/, and Epidemiology at http://www.epidem.com/). Information on the STROBE Initiative is available at http://www.strobe-statement.org.

For peer review only

BMJ Open

Cohort Profile: The Western Cape Pregnancy Exposure Registry (WCPER)

Journal:	*BMJ Open*
Manuscript ID	bmjopen-2021-060205.R2
Article Type:	Cohort profile
Date Submitted by the Author:	10-Jun-2022

Complete List of Authors:	Kalk, Emma; University of Cape Town Faculty of Health Sciences, Centre for Infectious Disease Epidemiology & Research<br>Heekes, Alexa; University of Cape Town Faculty of Health Sciences, Centre for Infectious Disease Epidemiology & Research; Western Cape Department of Health, Health Intelligence Directorate<br>Slogrove, Amy; Stellenbosch University, Ukwanda Centre for Rural Health, Department of Global Health; Stellenbosch University, Department of Paediatrics & Child Health<br>Phelanyane, Florence; University of Cape Town Faculty of Health Sciences, Centre for Infectious Disease Epidemiology & Research; Western Cape Department of Health, Health Intelligence Directorate<br>Davies, Mary-Ann; University of Cape Town Faculty of Health Sciences, Centre for Infectious Disease Epidemiology & Research; Western Cape Department of Health, Health Intelligence Directorate<br>Myer, Landon; University of Cape Town Faculty of Health Sciences, Division of Epidemiology & Biostatistics<br>Euvrard, Jonathan; University of Cape Town Faculty of Health Sciences, Centre for Infectious Disease Epidemiology & Research; Western Cape Department of Health, Health Intelligence Directorate<br>Kroon, Max; University of Cape Town Faculty of Health Sciences, Neonatology; Mowbray Maternity Hospital, Neonatal Services<br>Petro, Greg; University of Cape Town Faculty of Health Sciences, Department of Obstetrics & Gynaecology; New Somerset Hospital, Maternity Services<br>Fieggen, Karen; University of Cape Town Faculty of Health Sciences, Division of Human Genetics; Groote Schuur Hospital, Medical Genetics Services<br>Stewart, Chantal; University of Cape Town Faculty of Health Sciences, Department of Obstetrics & Gynaecology; Mowbray Maternity Hospital, Maternity Services<br>Rhoda, Natasha; University of Cape Town Faculty of Health Sciences, Department of Paediatrics and Child Health; Mowbray Maternity Hospital, Neonatal Services<br>Gebhardt, Stefan; Stellenbosch University, Department of Obstetrics & Gynaecology; Tygerberg Hospital, Maternity Services<br>Osman, Ayesha; University of Cape Town Faculty of Health Sciences, Department of Obstetrics & Gynaecology; Groote Schuur Hospital, Maternity Services<br>Anderson, Kim; University of Cape Town Faculty of Health Sciences, Centre for Infectious Disease Epidemiology & Research<br>Boulle, Andrew; University of Cape Town Faculty of Health Sciences,

	Centre for Infectious Disease Epidemiology & Research; Western Cape Department of Health, Health Intelligence Directorate<br>Mehta, Ushma; University of Cape Town Faculty of Health Sciences, Centre for Infectious Disease Epidemiology & Research
**Primary Subject Heading**:	Public health
Secondary Subject Heading:	Epidemiology, Health services research, Obstetrics and gynaecology, Pharmacology and therapeutics, Public health
Keywords:	PUBLIC HEALTH, EPIDEMIOLOGY, Maternal medicine < OBSTETRICS, Fetal medicine < OBSTETRICS

SCHOLARONE™
Manuscripts

I, the Submitting Author has the right to grant and does grant on behalf of all authors of the Work (as defined in the below author licence), an exclusive licence and/or a non-exclusive licence for contributions from authors who are: i) UK Crown employees; ii) where BMJ has agreed a CC-BY licence shall apply, and/or iii) in accordance with the terms applicable for US Federal Government officers or employees acting as part of their official duties; on a worldwide, perpetual, irrevocable, royalty-free basis to BMJ Publishing Group Ltd ("BMJ") its licensees and where the relevant Journal is co-owned by BMJ to the co-owners of the Journal, to publish the Work in this journal and any other BMJ products and to exploit all rights, as set out in our licence.

The Submitting Author accepts and understands that any supply made under these terms is made by BMJ to the Submitting Author unless you are acting as an employee on behalf of your employer or a postgraduate student of an affiliated institution which is paying any applicable article publishing charge ("APC") for Open Access articles. Where the Submitting Author wishes to make the Work available on an Open Access basis (and intends to pay the relevant APC), the terms of reuse of such Open Access shall be governed by a Creative Commons licence – details of these licences and which Creative Commons licence will apply to this Work are set out in our licence referred to above.

Other than as permitted in any relevant BMJ Author's Self Archiving Policies, I confirm this Work has not been accepted for publication elsewhere, is not being considered for publication elsewhere and does not duplicate material already published. I confirm all authors consent to publication of this Work and authorise the granting of this licence.

For peer review only

Cohort Profile: The Western Cape Pregnancy Exposure Registry (WCPER)

Authors: Emma Kalk[1*], Alexa Heekes[1,2,] Amy Slogrove[3,4], Florence Phelanyane[1,2], Mary-Ann Davies[1,2], Landon Myer[5], Jonathan Euvrard[1,2], Max Kroon[6,7], Greg Petro[8,9], Karen Fieggen[10,11], Chantal Stewart[8,12], Natasha Rhoda[6,7], Stefan Gebhardt[13,14], Ayesha Osman[8,15], Kim Anderson[1], Andrew Boulle[1,2†], Ushma Mehta[1†]

1. Centre for Infectious Disease Epidemiology & Research and Division of Public Health Medicine, School of Public Health & Family Medicine, University of Cape Town, South Africa

2. Health Intelligence Directorate, Western Cape Government Health, Cape Town, South Africa

3. Department of Paediatrics & Child Health, Faculty of Medicine & Health Sciences, Stellenbosch University, Worcester, South Africa

4. Ukwanda Centre for Rural Health, Department of Global Health, Faculty of Medicine & Health Sciences, Stellenbosch University, Worcester, South Africa

5. Division of Epidemiology & Biostatistics, School of Public Health & Family Medicine, University of Cape Town, South Africa

6. Department of Paediatrics and Child Health, University of Cape Town, South Africa

7. Mowbray Maternity Hospital, Neonatal Services, Cape Town, South Africa

8. Department of Obstetrics & Gynaecology, University of Cape Town, South Africa

9. New Somerset Hospital, Maternity Services, Cape Town, South Africa

10. Division of Human Genetics, University of Cape Town, Cape Town, South Africa

11. Groote Schuur Hospital, Medical Genetics Service, Cape Town, South Africa

12. Mowbray Maternity Hospital, Maternity Services, Cape Town, South Africa

13. Department of Obstetrics & Gynaecology, Stellenbosch University, Cape Town, South Africa

For peer review only - http://bmjopen.bmj.com/site/about/guidelines.xhtml

14. Tygerberg Hospital Maternity Services, Cape Town, South Africa

15. Groote Schuur Hospital, Maternity Services, Cape Town South Africa

*corresponding author: Centre for Infectious Disease Epidemiology & Research, School of Public Health & Family Medicine, 5th floor Falmouth Building, University of Cape Town Faculty of Health Sciences, Anzio Road, Observatory, Cape Town

emma.kalk@uct.ac.za

† Joint senior authors

For peer review only

ABSTRACT

Purpose: The Western Cape Pregnancy Exposure Registry (PER) was established at two public sector healthcare sentinel sites in the Western Cape province, South Africa to provide on-going surveillance of drug exposures in pregnancy and associations with pregnancy outcomes.

Participants: Established in 2016, all women attending their first antenatal visit at primary care obstetric facilities were enrolled and followed to pregnancy outcome regardless of the site (i.e., primary, secondary, tertiary facility). Routine operational obstetric and medical data are digitized from the clinical stationery at the health care facilities. Data collection has been integrated into existing services and information platforms and supports routine operations. The PER is situated within the Provincial Health Data Centre, an information exchange that harmonizes and consolidates all health-related electronic data in the province. Data are contributed via linkage across a unique identifier. This relationship limits the missing data in the PER, allows validation and avoids misclassification in the population-level dataset.

Findings to date: Approximately 5000 and 3500 pregnant women enter the dataset annually at the urban and rural sites, respectively. As of August 2021, >30 000 pregnancies have been recorded and outcomes have been determined for 93%. Analysis of key obstetric and neonatal health indicators derived from the PER are consistent with the aggregate data in the District Health Information System.

Future plans: This represents significant infrastructure, able to address clinical and epidemiological concerns in a low/middle-income setting.

Key words

Pregnancy Exposure Registry, Pharmacovigilance, Surveillance

For peer review only - http://bmjopen.bmj.com/site/about/guidelines.xhtml

Strengths and Limitations of this Study

The Western Cape Pregnancy Exposure Registry (PER) provides on-going surveillance of drug exposures in pregnancy and associations with pregnancy outcomes.

Data collection is integrated into existing services and information platforms and supports routine operations.

The PER is situated within the Provincial Health Data Centre, an information exchange that harmonizes and consolidates all health-related electronic data which limits missing data, allows validation and avoids misclassification in the population-level dataset.

The PER digitizes clinical data recorded in operational stationery and we cannot control for data quality nor account for missing data in the source documents nor for unmeasured confounders.

Medicines obtained outside the public sector systems and traditional and complementary medicines are not included unless they are documented in the clinical stationery.

For peer review only

For peer review only - http://bmjopen.bmj.com/site/about/guidelines.xhtml

Cohort Profile: The Western Cape Pregnancy Exposure Registry

Introduction

Assessing medicine and vaccine safety in pregnancy requires on-going surveillance across multiple settings. In high-income countries, reviews of outpatient prescriptions and self-medication during pregnancy estimated exposure rates of up to 93% and 43%, respectively, excluding vitamins and supplements[1, 2]. Reports from Africa, the site of mass prevention and treatment campaigns for HIV, tuberculosis and malaria, are less frequent: we estimate that 79% - 99% of women in Cape Town use medicines antenatally[3].

Pregnant women have been systematically excluded from pharmaceutical trials and the efficacy, dosing and safety of many medicines used during pregnancy are uncertain or findings delayed until after the product is licensed and in use. Post-authorization safety assessments have traditionally relied on passive reporting of suspected medicine-related adverse events. Such systems have been limited by their dependence on voluntary reporting, variable data quality, absence of background rates of adverse birth outcomes including common congenital disorders, and lack of data to establish a denominator.

Recently, pharmacovigilance in pregnancy has drawn public and political attention following concerns about the initial signal of potential association observed between the antiretroviral integrase inhibitor, dolutegavir, and neural tube defects[4, 5], the potential risk of isoniazid preventive therapy in women living with HIV[6] (WLHIV), and SARS-CoV-2 vaccines[7]. With all these exposures, synthesis and meta-analysis of the available data has been re-assuring and the World Health Organization (WHO) guidelines recommends no contra-indication to their use in pregnant and breast-feeding women [8-

For peer review only - http://bmjopen.bmj.com/site/about/guidelines.xhtml

For peer review only

10]. In addition, there have been increased calls globally for the inclusion of pregnant women in clinical trials for new therapeutic and preventive agents, particularly in the field of infectious disease[11-14].

Pregnancy Exposure Registries (PER) are a form of surveillance, designed to iteratively detect adverse events within a defined pregnant population. Importantly, the prospective nature of PER allows collection of exposure and other data before the pregnancy outcome is known. The pharmaceutical industry maintains drug-specific registries for medicines and/or drug classes with known/suspected teratogenic effects (e.g., Anti-Epileptic Drug Registries) or as part of post-marketing commitments (e.g., the Antiretroviral Pregnancy Register) [15, 16]. In addition, teratology information services may collect data on pregnancy exposures. These PER depend on voluntary enrolment by clinicians and/or women, and many do not directly collect data from comparator groups but rely either on internal comparators or on an identified external comparator to provide background prevalence data[17]. Background rates of adverse maternal and obstetric outcomes are necessary to determine deviations from expected proportions (signals). Such data may be limited or lacking in low- and middle-income countries[18, 19] or differ sufficiently from the source population so as to introduce bias (e.g., use of the Metropolitan Atlanta Congenital Defects Program as external comparator for USA-based studies[17].)

For peer review only

The UNICEF/UNDP/World Bank/WHO Special Programme for Research & Training in Tropical Diseases (TDR) has developed a PER approach for resource-limited settings aimed at prospective data collection on exposures in a cohort of pregnant women attending antenatal care services at sentinel sites. Important for validity and causality determination, the approach recommends inclusion of *all* women presenting to the site to allow concurrent establishment of background rates and assessment of multiple potential exposures[20].

For peer review only - http://bmjopen.bmj.com/site/about/guidelines.xhtml

The Western Cape (WC) PER was established in Cape Town in 2016, adapted from the TDR template. It was nested within the province-wide health information exchange, a component within a larger project designed to assess the impact of WHO Option B+ for vertical HIV transmission prevention (i.e., universal lifelong antiretroviral therapy [ART] for pregnant and breast-feeding women) at the population and individual levels[21]. Situating the PER within the linked information exchange avoided some of the limitations of exclusive primary-care databases in that both electronic inpatient and outpatient prescriptions are recorded as well as those from specialist and other off-site clinics, sources which may be absent from primary-care records[3, 22, 23]. The design also supports augmentation of the electronic clinical record for enrolled women, while providing a more secure, sustainable, and ethically-viable platform for capturing clinical data on mothers and infants.

We took a pragmatic approach to the establishment of the PER based on the availability of resources and the desire to integrate into existing systems and operational routines, avoiding a parallel infrastructure and supporting longevity. Data generated by the initiative are available for the evaluation and improvement of clinical care as well as epidemiological review.

For peer review only

Cohort description

The PER has been established at two sentinel sites in the WC. Gugulethu Midwife Obstetric Unit (GMOU) provides obstetric care to approximately 5000 women annually in Gugulethu, Cape Town a low-income area with high unemployment and an antenatal HIV prevalence of approximately 30%. GMOU refers patients to Mowbray Maternity (secondary) and Groote Schuur (tertiary) Hospitals. About half of all women who attend GMOU are referred to hospital, antenatally or perinatally. Worcester MOU (WMOU) is situated adjacent to the Worcester Provincial Hospital in Worcester, a

For peer review only - http://bmjopen.bmj.com/site/about/guidelines.xhtml

town of approximately 230 000 in a farming community 120 kilometers outside Cape Town. WMOU provides delivery services for ~3600 women annually. The antenatal HIV prevalence is approximately 16%. Women requiring more advanced care are referred to Worcester (secondary) and Tygerberg (tertiary) Hospitals. The community is structurally disadvantaged, and many depend on seasonal employment on farms. In both areas the population is mobile; women move within the WC province and may deliver outside the proscribed referral axes.

For peer review only

Enrolment started at GMOU in Cape Town in September 2016 and at WMOU in January 2018.

All women seeking care at the sentinel primary-care sites were included. Most women who use public maternity services, including those with medical and obstetric complications, initially present to primary care, therefore **situating enrolment at the primary-care facility allowed us to capture a sample representative of the pregnant population in the geographic drainage area of that facility.**

Maternal and Child Health Services in the Western Cape

Obstetric care is free at the point of service and approximately 65% of women present at/before 20 weeks gestation[24]. Antenatal care for uncomplicated pregnancies is provided at Basic Antenatal Clinics and MOUs, the latter able to manage uncomplicated vaginal deliveries. At any stage during pregnancy or peri-partum women can be referred to district, regional or tertiary hospitals according to standard operating procedures. HIV testing is routine at timepoints throughout gestation and WLHIV are initiated/re-initiated on ART[25]; those already receiving ART may transfer their HIV care to the MOU. Clients with other underlying medical conditions (e.g., pre-existing hypertension, diabetes mellitus, cardiac conditions) and/or who develop pregnancy-related medical conditions (e.g., hypertensive disorders of pregnancy, gestational diabetes) continue antenatal care at hospital. The

For peer review only - http://bmjopen.bmj.com/site/about/guidelines.xhtml

MOU dispenses ART and antenatal supplements and preventive therapies recommended by the WHO in pregnancy (i.e. iron and folate supplements, tetanus and influenza vaccines)[26]. Midwives treat the common complaints of pregnancy (heartburn, nausea), urinary tract infection, vaginal candidiasis and provide syndromic treatment for sexually transmitted infections (STI). Frequently, these medicines are dispensed directly from *ward-stock* without a linked digital record, although details are recorded in paper-based registers.

Within resource constraints, the WC endeavors to provide an antenatal ultrasound scan to clients before 22 weeks gestation for determining gestational age. If concerns are identified women are up-referred for formal fetal anomaly review.

Antenatal visits, HIV testing, transfers and deliveries are recorded against patient names in individual paper-based registers. Monthly aggregate statistics of key obstetric indicators (Table 1) are manually counted from these registers and submitted centrally as part of the routine District Health Information System platform.

Follow-up

The Maternity Case Record (MCR) is a patient-held paper-based document distributed at the first antenatal visit that serves as a record of all clinical obstetric care until discharge after pregnancy outcome, regardless of level of care. It is utilized throughout South Africa and archived at the site of outcome. Chronic medication and any agents dispensed during pregnancy should be recorded in the MCR by the attending clinicians. However, medicines received at specialist clinics, during hospital admissions and over-the-counter medicines are often not documented [3, 22].

For peer review only - http://bmjopen.bmj.com/site/about/guidelines.xhtml

For peer review only

After birth, live- and still-born neonates are examined by the attending clinician (nurse midwife/doctor) and the outcome of the limited neonatal surface examination is recorded in the MCR. This examination has been shown to detect most major congenital malformations in neonates, i.e. those that are visible and do not require diagnostic tools[27]. At GMOU, a clinician employed by the PER performs a review of clinical records to obtain additional data for congenital disorders and stillbirths. In the case of stillbirth, the placenta may be sent for histological examination.

In the WC, most women (99%) give birth at a health facility[24]. Those who do not, will bring their infants to the MOU soon after birth for review and registration.

For the purposes of the PER, the MCR serves as the primary source of prospectively-collected clinical data. Thus, women enter the cohort on first visit to the MOU and are followed up until pregnancy outcome.

Data collection

The PER digitizes routinely-collected data from the clinical stationery if not already digitized under existing service delivery. In addition to the patient-held MCR, data sources include primary-care dating ultrasound reports, and the STI and labour ward delivery registers. As we are using operational data, definitions have been aligned with operational clinical definitions in the WC. Using other routinely collected data elements (gestational age, neonate anthropometry) we are able to align case definitions with those of the Global Alignment of Immunization Safety Assessment in Pregnancy[28], allowing for harmonization of data and meaningful comparisons with equivalent datasets. Additionally, we collect or calculate health indicators for the routine monthly aggregate reports required by the MOUs (Table 1).

For peer review only - http://bmjopen.bmj.com/site/about/guidelines.xhtml

Externally-funded PER data clerks are embedded at the facilities and project-augmented data collection is accommodated within the routine patient and document flow without disruption of clinical care.

The provincial government of the WC operates as a single provider of public sector health services. A 9-digit numeric folder number which is common across the health platform for a given patient facilitates the harmonization of all electronic health records within the Provincial Health Data Centre (PHDC), the information exchange that consolidates all electronic administrative, pharmacy, laboratory, and disease-specific information[21]. PER data are recorded against this identifier and contribute to the PHDC.

All MOUs use the Primary Health Care Information Service (PHCIS) electronic medical records system which records attendance against patient identifiers, and ART in WLHIV. PHCIS automatically generates a unique folder number for live infants at birth, providing electronic linkage between mother and baby. Clinicom performs this function at all hospitals. Data are imported daily by the PHDC[21].

Completeness of Medicine Exposure data

Electronic dispensing data in the PHDC is augmented by the PER which captures medicine exposures elicited from the women during the clinical consultation and ward-stock medicines recorded by clinicians in the MCR. The PER also records some lifestyle factors (weight gain, alcohol, tobacco, recreational drugs) that may act as confounders for certain outcomes. Combining the electronic pharmacy data in the PHDC strengthens the ascertainment of exposures, providing a complete list of medication dispensed from public sector pharmacies. Using multiple data sources for this has been

For peer review only - http://bmjopen.bmj.com/site/about/guidelines.xhtml

shown to provide a more complete picture of antenatal medicine use essential for pregnancy exposure research[3, 29, 30].

Outcome Ascertainment

Information on neonatal outcomes such as vital status, birth weight, gestational age, and APGAR scores tend to be consistently captured across the cohort. The key findings of the neonatal surface examinations, although often perfunctory, usually result in the recording of notable physical anomalies. Internal anomalies such as cleft palate, hip dysplasias, and cardiac anomalies as well as more subtle dysmorphic features may be missed at the time of the initial neonatal examination. Details of neonatal deaths, and major congenital disorders often require review of inpatient records at the delivery facilities.

PER data are imported daily into the PHDC and linked using patient identifiers, providing a comprehensive electronic clinical record at the level of the individual which is accessible to the attending clinicians. Both systems benefit greatly from this design. The PER allows for validation of the provincial dataset as relates to pregnancy and delivery, and the PHDC is able to identify missing outcomes (often at sites outside the referral axes) or exposures (from electronic pharmacy dispensing) not included in the PER.

<u>Findings to date</u>

Between 01 September 2016 and 31 August 2021, 31 346 pregnancies were recorded in the PER. To assess robustness of the dataset, we analysed data for a subset of women who attended their first visit to antenatal care between 01 January 2018 and 31 December 2019 (Table 2). Over this two-year period, 14 527 individual pregnancies were recorded in the PER: 9435 and 5092 at the urban and rural

For peer review only - http://bmjopen.bmj.com/site/about/guidelines.xhtml

site, respectively. Outcomes were determined for 93.4% of pregnancies (n = 13 574). Gestational dating scans were performed in 38.5% (n = 5583) of all enrolees, of whom 60% (n = 3345) were ≤22 weeks, facilitating more precise gestational dating at birth as well as timing of exposures. Overall, 1287 women (9%) were exposed to a medicines with pregnancy safety surveillance requirements (Table 3 and Supplementary Table 1).

Where relevant, we compared rates of key adverse birth outcomes in the PER with official aggregate routine indicator data for the WC [24, 31-33], derived from register aggregates reported through the District Health Information System (DHIS) (Table 3). The comparisons are re-assuring across both the urban and rural sites, validating the indicator outputs of the PER and demonstrating utility to the services. The data will contribute to detailed aggregate reports for facility managers and streamline the monthly submissions to the DHIS which are currently based on manual counts.

The congenital disorder data are still being cleaned for analysis with pregnancy outcomes.

Published and other outputs

We conducted an initial baseline assessment comparing clinical records to dispensing data before the implementation of the PER[22] and recently updated the analysis demonstrating the value of combining PER and electronic pharmacy data in improving medicine exposure ascertainment[3]. We are currently investigating the impact of data source on gestational age (Malaba T, manuscript in preparation) and hypertensive disorders of pregnancy[34]. PER data have contributed to population-based analyses describing the use and safety of sodium valproate and isoniazid for TB preventive therapy in pregnancy[35, 36]. In addition, initiation of the PER provided the opportunity to host a workshop, *Building Teratovigilance Capacity in Africa,* which provided networking and training

For peer review only - http://bmjopen.bmj.com/site/about/guidelines.xhtml

opportunities to 60 delegates from sub-Saharan Africa https://globalpharmacovigilance.tghn.org/resources/building-teratovigilance-capacity-africa/.

System strengthening

In addition to the employment of project-specific staff, embedded with computers at the facilities, the project supports on-going training of clinical staff to improve and standardize clinical history-taking with an emphasis on exposures, neonatal examination and clinical record keeping. Open resources include the WHO/TDR *Stepwise Surface Examination of the Newborn* (https://www.who.int/tdr/publications/videos/stepwise-surface-examination-newborns/en/) and the training modules for midwives we developed as part of the South African National Pregnancy Exposure Registry (https://www.ubomibuhle.org.za/training-lessons)[37]. These resources are freely available and are now in use at PER sites across South Africa.

Strengths and Weaknesses

Strengths

The integration of the PER within the PHDC greatly increases the completeness of the data. It facilitates identification of pregnancy outcomes at facilities outside our sentinel referral chains reducing loss to follow-up. Harmonization and triangulation of two data sources for medicine exposures (i.e., clinical records and electronic pharmacy records) provides a more robust summary of exposures than either alone[3, 17, 22]. These systems comprise unique infrastructure able to address clinical and public health concerns in a low-middle income setting.

For peer review only - http://bmjopen.bmj.com/site/about/guidelines.xhtml

Accurate timing of exposures over the course of pregnancy is crucial to assess potential associations with adverse pregnancy outcomes. Collecting multiple reference points for gestational age (i.e., neonatal record, ultrasound, last menstrual period, symphysis-fundal height) enabled the development of a hierarchy of methods and the allocation of a confidence score to the reported gestational age[38-40]. This offers an advantage over insurance claims datasets which are often used to determine safety information and in which pregnancy and gestational age must be inferred from clinical coding alone.

In line with the TDR protocol [20], all women attending the PER primary care sites are enrolled and we reflect background rates of important pregnancy parameters similar to what is expected from national aggregate data. This will be expanded to include background rates for congenital disorders, data which are lacking in South Africa[41]. This structure also allows for the analysis of multiple current and potential future exposures and emerging health concerns e.g., novel medicines and vaccines such as for SARS-CoV-2. Determining the rates and associations of rare events such as major congenital anomalies requires large, representative samples. Such analyses necessitate resources for data cleaning and interpretation, especially to determine the timing of drug/teratogen exposures over the course of gestation. This work is currently underway in the PER.

From the outset, it was important to avoid a parallel system and support project sustainability. The PER has been integrated into the existing clinical and clerical routines and uses local electronic health information platforms. It allows for electronic generation of key monthly indicators at primary care sites that are otherwise collected by hand.

For peer review only - http://bmjopen.bmj.com/site/about/guidelines.xhtml

For peer review only

As the cohort expands, capacity to conduct nested studies that facilitate signal detection and signal verification of potential or suspected teratogens will improve. The collection of individual-level data in a large prospectively enrolled cohort, representative of both urban and rural WC populations who use public sector services will support more robust analyses that can better account for confounding factors in such observational data.

Weaknesses

The PER digitizes clinical data recorded in operational stationery and we cannot control for data quality nor account for missing data in the source documents, including the risk of under-reporting. To address this, we have engaged in on-going training at the sites with an emphasis on drug history taking, medical record-keeping and neonatal examination offering in-person teaching and video tutorials. Clinical staff have been provided with Medicine Identification Aids with photographs of common formulations and packaging, and the WHO Birth Defects atlas[42]. However, misclassification remains a potential risk.

Notwithstanding the advantages of the individual-level data available within the PHDC, data are limited to those that are entered into one of the electronic medical records systems used in the public sector. In terms of medicine exposures, the PER documents dispensed medication which may not reflect actual use. In addition, medicines obtained outside of the public sector systems, from private doctors or over-the-counter from pharmacies are not included unless they are noted in the clinical records[3]. Similarly, traditional and complementary medicines lack a linked electronic footprint and are not included.

For peer review only - http://bmjopen.bmj.com/site/about/guidelines.xhtml

The PER database is parsimonious by design and necessity and we are unable to account for unmeasured confounders. However, data fields are collected for the entire cohort who are all drawn from the same geographical areas served by the primary care clinics. Additionally, we record limited data on lifestyle factors relevant in pregnancy (weight gain, exposure to tobacco, alcohol, recreational drugs) which are lacking from equivalent population datasets based on insurance claims data.

Collaboration

As with the PHDC within which it is located, the PER can address clinical, operational and research needs, and data access is specific to each. Aggregate reports are available to managers. Data are anonymised using standard protocols for de-identifying records before they are shared with researchers who are not directly engaged in the women's clinical care. It is anticipated that such de-identified individual-level data may be shared as part of the South African National Pregnancy Registry[37] and with similar PER initiatives regionally or internationally[43]. Data-sharing commitments are particularly relevant to research of rare events such as congenital disorders[20]. The PHDC has in-built privacy systems and strict governance structures managing the protection and use of health data for both service and research purposes and these apply to the PER[21].

Patient and public involvement

The PER is integrated into the data collection and curation services of the Western Cape Government Department of Health and clinical and other service providers have engaged with the project since its inception. The data are available to managers as aggregate reports and to contribute to the electronic clinical records accessible by clinicians. Feedback from users contributes iteratively to optimization of the PER to improve health outcomes for pregnant women and infants.

For peer review only

Conclusions

Research on medicine safety in pregnancy requires data on individual pregnancies, mother-infant linkage, medication exposure, gestational age at exposure, and maternal and birth outcomes. Data completeness and robustness continues to improve with on-going training, evolution of routine clinical information systems, and increasing political focus on pregnancy exposures. The cohort is well-placed to detect large signals in pregnancy outcomes as novel maternal exposures are introduced, and to contribute to cohort harmonization for rarer outcomes and address the lack of information on congenital disorders in Africa.

Ethics approval

The WC PER has been approved by the Faculty of Health Sciences Human Research Ethics Committees of the University of Cape Town (HREC: 749/2015) and Stellenbosch University (N17/04/040, N20/08/084), and the Western Cape Government Health Research Committee. The requirement for individual informed consent was waived by the ethics committee and all data were anonymized before being transferred for analysis.

Funding

This work was supported by the US National Institutes of Health [R01HD080465, U01AI069911, U01AI069924, K43TW010683], Bill and Melinda Gates Foundation [INV-004508; 1164272; 1191327; INV-004657], the Wellcome Trust [203135/Z/16/Z], the International AIDS Society [2017-518/SLO], the United States Agency for International Development [72067418CA00023] and the US Centers for Disease Control and Prevention [GH001934].

For peer review only - http://bmjopen.bmj.com/site/about/guidelines.xhtml

Acknowledgements

We acknowledge and thank the PER data teams in Cape Town and Worcester, and the clinical and management staff at the facilities. The support and investment of the subdistrict and provincial health management is crucial to maintaining the initiative. We further acknowledge colleagues from the Western Cape Government: Health and the City of Cape Town.

Author contributions

Conception and design: EK, UM, AB, ALS, MAD, LM

Design and Implementation including data systems: EK, UM, ALS, GP, KF, JE

Data harmonization: AH, FP, JE, AB, MAD, EK

Clinical oversight: GP, MK, CS, NR, SG, AO, KF

Data cleaning & analysis: EK, ALS, UM, KA, AH

All authors critically reviewed the manuscript.

Conflict of interests

EK, AB, MAD and KA received funding from Viiv Healthcare unrelated to this project.

The authors declare no conflicts of interest.

For peer review only

References

1. Daw JR, Hanley GE, Greyson DL, Morgan SG. Prescription drug use during pregnancy in developed countries: a systematic review. Pharmacoepidemiol Drug Saf. 2011;20(9):895-902.
2. Mohseni M, Azami-Aghdash S, Gareh Sheyklo S, Moosavi A, Nakhaee M, Pournaghi-Azar F, et al. Prevalence and Reasons of Self-Medication in Pregnant Women: A Systematic Review and Meta-Analysis. Int J Community Based Nurs Midwifery. 2018;6(4):272-84.
3. van der Hoven J, Allen E, de Waal R, Cois A, Maartens G, Myer L, et al. Antenatal medicine use in South African women: a comparison of three methods of ascertainment. BMC Pregnancy Childbirth. 2022;22(1):1-15
4. Zash R, Holmes L, Diseko M, Jacobson DL, Brummel S, Mayondi G, et al. Neural-Tube Defects and Antiretroviral Treatment Regimens in Botswana. N Engl J Med. 2019;381(9):827-40.
5. Zash R, Makhema J, Shapiro RL. Neural-Tube Defects with Dolutegravir Treatment from the Time of Conception. N Engl J Med. 2018;379(10):979-81.
6. Gupta A, Montepiedra G, Aaron L, Theron G, McCarthy K, Bradford S, et al. Isoniazid Preventive Therapy in HIV-Infected Pregnant and Postpartum Women. N Engl J Med. 2019;381(14):1333-46.
7. Bianchi DW, Kaeser L, Cernich AN. Involving Pregnant Individuals in Clinical Research on COVID-19 Vaccines. JAMA. 2021;325(11):1041-2.
8. World Health Organization. Module 1: Prevention, Tuberculosis Preventive Treatment in *Consolidated Guidelines on Tuberculosis* Geneva: World Health Organization; 2020 [Available from: https://tbksp.org/en/node/43 .] (Accessed 17 March 2022)
9. World Health Organization. CONSOLIDATED GUIDELINES ON HIV PREVENTION, TESTING, TREATMENT, SERVICE DELIVERY AND MONITORING: RECOMMENDATIONS FOR A PUBLIC HEALTH APPROACH Geneva: World Health organization; 2021 [Available from: https://www.who.int/publications/i/item/9789240031593.] (Accessed 17 March 2022)
10. World Health Organization. COVID-19 vaccines: safety surveillance manual, second edition Geneva: World Health Organization; 2021 [Available from: https://www.who.int/publications/i/item/10665345178.] (Accessed 17 March 2022)
11. Craig AM, Hughes BL, Swamy GK. Coronavirus disease 2019 vaccines in pregnancy. Am J Obstet Gynecol MFM. 2021;3(2):100295.
12. Gupta A, Hughes MD, Garcia-Prats AJ, McIntire K, Hesseling AC. Inclusion of key populations in clinical trials of new antituberculosis treatments: Current barriers and recommendations for pregnant and lactating women, children, and HIV-infected persons. PLoS Med. 2019;16(8):e1002882.
13. Moodley J, Ngene NC, Khaliq OP, Hunter M. An imperative to offer pregnant and lactating women access to the COVID-19 vaccination roll-out programme. S Afr Med J. 2021;111(6):567-9.
14. World Health Organization, International Maternal Pediatric Adolescent AIDS Clinical Trials Network. APPROACHES TO ENHANCE AND ACCELERATE STUDY OF NEW DRUGS FOR HIV AND ASSOCIATED INFECTIONS IN PREGNANT WOMEN. Geneva: World Health Organization; 2021. [https://www.who.int/publications/i/item/9789240040182]. (Accessed 17 March 2022)
15. Holmes LB, Wyszynski DF. North American antiepileptic drug pregnancy registry. Epilepsia. 2004;45(11):1465.
16. Scheuerle A, Covington D. Clinical review procedures for the Antiretroviral Pregnancy Registry. Pharmacoepidemiol Drug Saf. 2004;13(8):529-36.
17. Gelperin K, Hammad H, Leishear K, Bird ST, Taylor L, Hampp C, et al. A systematic review of pregnancy exposure registries: examination of protocol-specified pregnancy outcomes, target sample size, and comparator selection. Pharmacoepidemiol Drug Saf. 2017;26(2):208-14.
18. Berrueta M, Ciapponi A, Bardach A, Cairoli FR, Castellano FJ, Xiong X, et al. Maternal and neonatal data collection systems in low- and middle-income countries for maternal vaccines active safety surveillance systems: A scoping review. BMC Pregnancy Childbirth. 2021;21(1):217.
19. Mehta U, Kalk E, Fairlie L, Boulle A, Rees H. Why South Africa urgently needs to support the development of pregnancy exposure registries. S Afr Med J. 2019;109(5):294-5.

For peer review only - http://bmjopen.bmj.com/site/about/guidelines.xhtml

20. Mehta U, Clerk C, Allen E, Yore M, Sevene E, Singlovic J, et al. Protocol for a drugs exposure pregnancy registry for implementation in resource-limited settings. BMC Pregnancy Childbirth. 2012;12:89.
21. Boulle A, Heekes A, Tiffin N, Smith M, Mutemaringa T, Zinyakatira N, et al. Data Centre Profile: The Provincial Health Data Centre of the Western Cape Province, South Africa. Int J Popul Data Sci. 2019;4(2):1143.
22. Mehta U, Heekes A, Kalk E, Boulle A. Assessing the value of Western Cape Provincial Government health administrative data and electronic pharmacy records in ascertaining medicine use during pregnancy. S Afr Med J. 2018;108(5):439-43.
23. Okoli GN, Myles P, Murray-Thomas T, Shepherd H, Wong ICK, Edwards D. Use of Primary Care Data in Research and Pharmacovigilance: Eight Scenarios Where Prescription Data are Absent. Drug Saf. 2021;44(10):1033-1040.
24. Day C, Gray C, Padayachee T, Cois A. Chapter 20, Health and Related Indicators. South African Health Review 2020. 2020.
25. South African Department of Health. Guideline for the Prevention of Mother to Child Transmission of Communicable Infections. In: Health, editor. Pretoria. November 2019. [Available from: https://www.nicd.ac.za/wp-content/uploads/2019/11/Guidelines-for-the-Prevention-of-Transmission-of-Communicable-Diseases-from-mother-to-child_28-October.pdf]
26. World Health Organization. WHO recommendations on antenatal care for a positive pregnancy experience. Geneva: WHO Library Cataloguing-in-Publication Data; 2016. [Available from: https://www.who.int/publications/i/item/9789241549912]
27. Holmes LB, Nasri HZ, Hunt AT, Zash R, Shapiro RL. Limited surface examination to evaluate potential teratogens in a resource-limited setting. Birth Defects Res. 2021;113(9):702-7.
28. Bonhoeffer J, Kochhar S, Hirschfeld S, Heath PT, Jones CE, Bauwens J, et al. Global alignment of immunization safety assessment in pregnancy - The GAIA project. Vaccine. 2016;34(49):5993-7.
29. Andrade SE, Berard A, Nordeng HME, Wood ME, van Gelder M, Toh S. Administrative Claims Data Versus Augmented Pregnancy Data for the Study of Pharmaceutical Treatments in Pregnancy. Curr Epidemiol Rep. 2017;4(2):106-16.
30. Grzeskowiak LE, Gilbert AL, Morrison JL. Investigating outcomes associated with medication use during pregnancy: a review of methodological challenges and observational study designs. Reprod Toxicol. 2012;33(3):280-9.
31. Massyn N, Day C, Ndlovu N, Padayachee T. District Health Barometer 2019/2020. Durban: Health Systems Trust; 2020. [Available from: https://www.hst.org.za/publications/District%20Health%20Barometers/DHB%202019-20%20Complete%20Book.pdf]
32. National Perinatal Mortality and Morbidity Committee. NAPEMMCO Trennial Report 2017 - 2019. 2017-2019.
33. Provincial Perinatal Morbidity and Mortality Commitee. Western Cape Saving Babies Trennial Report 2017 -2019. 2020.
34. Slogrove A, Davies MA, Williams P, Abrams E, Theron G, Cotton M, et al., editors. Hypertensive Disorders in Pregnancy and HIV: A Province-wide Cohort Analysis during 2018 and 2019 in the Western Cape, South Africa. 13th International Workshop on HIV Pediatrics; 2021 16-17 July 2021; virtual.
35. Kalk E, Heekes A, Mehta U, de Waal R, Jacob N, Cohen K, et al. Safety and Effectiveness of Isoniazid Preventive Therapy in Pregnant Women Living with Human Immunodeficiency Virus on Antiretroviral Therapy: An Observational Study Using Linked Population Data. Clin Infect Dis. 2020;71(8):e351-e8.
36. Mehta U, Smith M, Kalk E, Hayes H, Swart A, Tucker L, et al. Understanding and Responding to Prescribing Patterns of Sodium Valproate-Containing Medicines in Pregnant Women and Women of Childbearing Age in Western Cape, South Africa. Drug Saf. 2021;44(1):41-51.
37. Ubomi Buhle, Understanding Birth Outcomes for Mothers and Infants: Building Healthcare by Linking Exposures 2021 [Available from: https://www.ubomibuhle.org.za/.]

For peer review only - http://bmjopen.bmj.com/site/about/guidelines.xhtml

38. Geerts L, Poggenpoel E, Theron G. A comparison of pregnancy dating methods commonly used in South Africa: a prospective study. S Afr Med J. 2013;103(8):552-6.
39. Malaba TR, Newell ML, Madlala H, Perez A, Gray C, Myer L. Methods of gestational age assessment influence the observed association between antiretroviral therapy exposure, preterm delivery, and small-for-gestational age infants: a prospective study in Cape Town, South Africa. Ann Epidemiol. 2018;28(12):893-900.
40. Quinn JA, Munoz FM, Gonik B, Frau L, Cutland C, Mallett-Moore T, et al. Preterm birth: Case definition & guidelines for data collection, analysis, and presentation of immunisation safety data. Vaccine. 2016;34(49):6047-56.
41. Lebese V, Aldous C, Malherbe HL. South African congenital disorders data, 2006 - 2014. S Afr Med J. 2016;106(10):992-5.
42. WHO/CDC/ICBDSR. Birth defects surveillance: atlas of selected congenital anomalies. Geneva: World Health Organization; 2015. [Available from: https://apps.who.int/iris/handle/10665/127941]
43. International epidemiology Databases to Evaluate AIDS (IeDEA)-East Africa. Measuring Adverse Pregnancy & Newborn Outcomes (MANGO): an IedEA Collaboration Study 2021 [Available from: https://globalhealth.iu.edu/research/iedea-main-page/.]

For peer review only

For peer review only - http://bmjopen.bmj.com/site/about/guidelines.xhtml

Table 1. Summary of Data Elements in the PER

Variables collected	Calculated parameters	MOU aggregate statistics
Antenatal<br>Maternal date of birth<br>Date of first antenatal visit<br>Last menstrual period<br>Parity, Gravidity<br>Obstetric & Medical history<br>Chronic medication<br>Height, mid-upper arm circumference, weight, blood pressure, urinalysis<br>Symphysis fundal height<br>Alcohol, tobacco, drug use<br>Number of antenatal visits	Gestational age at first antenatal visit	Number of first visits<br>Number of women first ANC < 20 weeks<br>Number age < 20 years or > 38 years<br>Number grand multipara (≥ 5 deliveries)<br>Number high blood pressure/proteinuria
Vertical transmission of HIV<br>HIV status at first antenatal visit<br>Subsequent positive HIV test<br>HIV treatment incl. regimen switches<br>CD4 count<br>Viral load<br>HIV-exposed infant HIV-PCR	Number of women at high risk of vertical HIV transmission<br>ART in hand at estimated time of conception<br>ART in hand at delivery	Number of women living with HIV:<br>Before pregnancy<br>During pregnancy<br>Number of women on ART (1st & 2nd line):<br>Before, during pregnancy<br>VL unsuppressed at pregnancy & delivery<br>Number of infant birth HIV-PCR
Ultrasound<br>Gestational age<br>Abnormalities<br>Expected date of delivery		Number of ultrasounds conducted<br>Number multiple pregnancies
Maternal outcome<br>Facility-based death	Vital status	Maternal death
Peri-partum<br>Date & site of outcome		Number of deliveries

For peer review only

For peer review only - http://bmjopen.bmj.com/site/about/guidelines.xhtml

Method of delivery<br>Gestational age at outcome	Prematurity (< 37 completed weeks gestation)	
Pregnancy outcome<br>Livebirth<br>Stillbirth<br>Miscarriage<br>Termination of pregnancy<br>Ectopic pregnancy<br>Molar pregnancy	Gestational age at pregnancy outcome	Number of livebirths, stillbirths, miscarriages
Neonate<br>Date of birth<br>Sex, APGAR scores<br>Gestational age<br>Birth weight, length, head circumference, foot length<br>Neonatal surface examination<br>Abnormalities noted	Gestational age at birth<br>Low birth weight (< 2500g)<br>Prematurity (< 37 completed weeks gestation)<br>Neonatal death	Number of low birth weight infants<br>Number premature infants<br>Number neonatal deaths<br>Perinatal mortality rate

ANC – antenatal care; ART – antiretroviral therapy; HIV – Human Immune Deficiency Virus; MOU – midwife obstetric unit; PCR – polymerase chain reaction

For peer review only

For peer review only - http://bmjopen.bmj.com/site/about/guidelines.xhtml

Table 2. Maternal and obstetric characteristics of the cohort 2018-2019

Variable	PER total n = 14 527	PER urban n = 9435 (65%)	PER rural n = 5092 (35%)
Age (years) median (IQR)	27 (23 – 32)	28 (23 – 33)	26 (22 – 31)
Living with HIV at pregnancy outcome	3931 (27.1)	3241 (34.3)	690 (13.6)
Obstetric ultrasound present n (%)<br>Early ultrasound (i.e. < 22 weeks) n (% of US)	5583 (38.4)<br>3345 (59.9)	4063 (43.1)<br>2393 (58.9)	1520 (29.9)<br>952 (62.6)
Potentially unsafe medicine exposure	1287 (9.0)	857 (9.3)	430 (8.5)
Gestational age at birth (weeks) median (IQR)	40 (37 – 40)	40 (36 – 40)	39 (35 – 40)
Birth weight (grams) median (IQR)	3100 (2750 – 3440)	3140 (2800 – 3480)	2975 (2575 – 3320)
Low birth weight[a] n (%)	1736 (12.0)	879 (9.3)	857 (16.8)
Premature birth[b] n(%)	2949 (20.3)	1735 (18.4)	1214 (23.8)
Pregnancy outcome n (%)			
Live birth	12 419 (85.5)	1189 (82.3)	4630 (90.9)
Still birth	296 (2.0)	180 (1.9)	116 (2.3)
Neonatal death[c]	109 (0.8)	71 (0.5)	36 (0.7)
Miscarriage	395 (2.7)	318 (3.4)	77 (1.5)
Ectopic pregnancy	82 (0.6)	60 (0.6)	22 (0.4)
Termination of pregnancy	273 (1.9)	223 (2.4)	50 (1.0)
Unknown	953 (6.6)	792 (8.4)	161 (3.1)
Delivery method[d] n(%)			
Born before arrival at birthing facility	608 (4.7)	245 (3.1)	363 (7.6)
Vaginal delivery	7587 (59.2)	4655 (57.9)	2932 (61.3)
Assisted delivery[e]	140 (1.1)	51 (0.6)	89 (1.9)
Caesarean section	3416 (26.6)	2411 (30.0)	1005 (21.0)
Unknown	1073 (8.4)	680 (8.5)	393 (8.2)
Infant outcome[d] n(%)			
Stillborn	296 (2.3)	180 (2.2)	116 (2.4)
Early neonatal death[c]	80 (0.6)	55 (0.7)	25 (0.5)
Late neonatal death	29 (0.2)	18 (0.2)	11 (0.2)
Alive	12 419 (96.8)	7798 (96.9)	4630 (96.8)
Tobacco use[f] n(%)			
Current user	1297 (8.9)	87 (0.9)	1210 (23.8)
Past user	55 (0.4)	13 (0.1)	42 (0.8)
Never user	9997 (68.8)	7222 (76.5)	2775 (54.5)
Not reported	3178 (21.9)	2113 (14.5)	1065 (7.3)
Alcohol use[f] n(%)			
Current user	588 (4.1)	339 (3.6)	249 (4.9)
Past user	167 (1.2)	66 (0.7)	101 (2.0)
Never user	10 570 (72/8)	6885 (73.0)	3685 (72.4)
Not reported	3202 (22.0)	2145 (14.8)	1057 (7.3)

[a] birthweight<2500g; liveborn infants only

[b] birth < 37 completed weeks gestation; liveborn infants only

For peer review only - http://bmjopen.bmj.com/site/about/guidelines.xhtml

[c] neonatal death: death before 28 days of life; early neonatal death: death before 7days of life; late neonatal death: death between 8 and 28 days of life

[d] viable pregnancies (i.e >27 weeks gestation(17)) (n=12 824)

[e] forceps or vacuum delivery

[f] reported at first antenatal visit

For peer review only

For peer review only - http://bmjopen.bmj.com/site/about/guidelines.xhtml

Table 3. Comparison between PER reported or calculated PER outcomes and aggregate indicators in formal provincial information systems

Indicator	PER total n = 14 527	PER urban n = 9435 (65%)	PER rural n = 5092 (35%)	Reported aggregate for the Western Cape 2017 - 2019[a]
Still birth[b] n (%)<br><br>Per 1000 births	296 (2.0)<br><br>20.0	180 (1.9)<br><br>19.1	116 (2.4)<br><br>24.0	2.2%[33]<br><br>18.5[31]<br>22.1[31-32]
Neonatal death in facility rate[c] per 1000 live births	8.7	9.2	7.7	8.9[31-32]
Perinatal mortality rate[d] per 1000 births	29	29	29	25.6[31]<br>27.9[32]<br>29.1[33]
Low birth weight[e] n(%)	1737 (12.0)	879 (9.3)	857 (16.8)	14.9% urban subdistrict<br>18.4% rural subdistrict[33]
Maternal mortality in facility ratio per 100 000 live births		63.5	Insufficient data	43.6 – 66.8[32]
Teenage pregnancies (10 – 19 years) n(%)	929 (6.4)	450 (4.8)	497 (9.4)	3.5% urban subdistrict<br>7.3% rural subdistrict[33]
Caesarean section rate per 1000 births	3416 (26.6)	2411 (30.0)	1005 (21.0)	28.9[32] - 29.3[33]

[a] includes aggregate reports compiled from the District Health Information System and Perinatal Problem Identification Programme[31-33]

[b] delivery of a baby with no signs of life after 27 completed weeks of gestation (i.e., viable baby born dead)

[c] death before 28 days of life

[d] still birth plus neonatal deaths <8 days per 1000 births

[e] birthweight<2500g; liveborn infants only

For peer review only

For peer review only

For peer review only - http://bmjopen.bmj.com/site/about/guidelines.xhtml

Supplementary Table 1. Potentially unsafe medicines, excluding ART, identified in the PER over the course of gestation (alphabetical)

Name
Carbamazepine
Carbimazole
Diazepam
Doxycycline
Enalapril
Gentamicin
Ibuprofen
Lithium
Losartan
Phenytoin
Sulfamethoxazole & trimethoprim
Valproate
Warfarin

ART – antiretroviral therapy

For peer review only

STROBE Statement—Checklist of items that should be included in reports of ***cohort studies***

	Item No	Recommendation	page
**Title and abstract**	1	(*a*) Indicate the study's design with a commonly used term in the title or the abstract	1
		(*b*) Provide in the abstract an informative and balanced summary of what was done and what was found	3
**Introduction**			
Background/rationale	2	Explain the scientific background and rationale for the investigation being reported	5-7
Objectives	3	State specific objectives, including any prespecified hypotheses	7
**Methods**			
Study design	4	Present key elements of study design early in the paper	7-8
Setting	5	Describe the setting, locations, and relevant dates, including periods of recruitment, exposure, follow-up, and data collection	7 - 10
Participants	6	(*a*) Give the eligibility criteria, and the sources and methods of selection of participants. Describe methods of follow-up	8-9
		(*b*) For matched studies, give matching criteria and number of exposed and unexposed	N/A
Variables	7	Clearly define all outcomes, exposures, predictors, potential confounders, and effect modifiers. Give diagnostic criteria, if applicable	11-12, 16
Data sources/ measurement	8*	For each variable of interest, give sources of data and details of methods of assessment (measurement). Describe comparability of assessment methods if there is more than one group	11-12 Table 1
Bias	9	Describe any efforts to address potential sources of bias	14-16
Study size	10	Explain how the study size was arrived at	N/A
Quantitative variables	11	Explain how quantitative variables were handled in the analyses. If applicable, describe which groupings were chosen and why	12-13 Tables 2-4
Statistical methods	12	(*a*) Describe all statistical methods, including those used to control for confounding	Cohort *description;* limited analyses
		(*b*) Describe any methods used to examine subgroups and interactions	N/A
		(*c*) Explain how missing data were addressed	14-16
		(*d*) If applicable, explain how loss to follow-up was addressed	16
		(*e*) Describe any sensitivity analyses	12-13
**Results**			
Participants	13*	(a) Report numbers of individuals at each stage of study—eg numbers potentially eligible, examined for eligibility, confirmed eligible, included in the study, completing follow-up, and analysed	12-13
		(b) Give reasons for non-participation at each stage	N/A
		(c) Consider use of a flow diagram	
Descriptive data	14*	(a) Give characteristics of study participants (eg demographic, clinical, social) and information on exposures and potential confounders	12-13 Tables 2-4
		(b) Indicate number of participants with missing data for each variable of interest	N/A
		(c) Summarise follow-up time (eg, average and total amount)	N/A
Outcome data	15*	Report numbers of outcome events or summary measures over time	12-13

For peer review only - http://bmjopen.bmj.com/site/about/guidelines.xhtml

			Tables 2-4
Main results	16	(*a*) Give unadjusted estimates and, if applicable, confounder-adjusted estimates and their precision (eg, 95% confidence interval). Make clear which confounders were adjusted for and why they were included	N/A
		(*b*) Report category boundaries when continuous variables were categorized	N/A
		(*c*) If relevant, consider translating estimates of relative risk into absolute risk for a meaningful time period	
Other analyses	17	Report other analyses done—eg analyses of subgroups and interactions, and sensitivity analyses	N/A
**Discussion**			
Key results	18	Summarise key results with reference to study objectives	13-14
Limitations	19	Discuss limitations of the study, taking into account sources of potential bias or imprecision. Discuss both direction and magnitude of any potential bias	16
Interpretation	20	Give a cautious overall interpretation of results considering objectives, limitations, multiplicity of analyses, results from similar studies, and other relevant evidence	17-18
Generalisability	21	Discuss the generalisability (external validity) of the study results	14, 17- 18
**Other information**			
Funding	22	Give the source of funding and the role of the funders for the present study and, if applicable, for the original study on which the present article is based	18

*Give information separately for exposed and unexposed groups.

Note: An Explanation and Elaboration article discusses each checklist item and gives methodological background and published examples of transparent reporting. The STROBE checklist is best used in conjunction with this article (freely available on the Web sites of PLoS Medicine at http://www.plosmedicine.org/, Annals of Internal Medicine at http://www.annals.org/, and Epidemiology at http://www.epidem.com/). Information on the STROBE Initiative is available at http://www.strobe-statement.org.

For peer review only

For peer review only - http://bmjopen.bmj.com/site/about/guidelines.xhtml
